# Up-Regulation of the TRPM8 Channel Attenuates TRPC1-Mediated Store-Operated Calcium Entry in Abdominal Aortic Aneurysm

**DOI:** 10.3390/biom16050741

**Published:** 2026-05-19

**Authors:** Yi-Qian Wang, Min Pan, Yi-Chen Lin, Si-Yi Zheng, Qin-Ye Chen, Long-Xin Gui, Mo-Jun Lin, Da-Cen Lin

**Affiliations:** 1 Department of Physiology and Pathophysiology, School of Basic Medical Sciences, Fujian Medical University, Fuzhou 350122, China; yiqianwang@fjmu.edu.cn (Y.-Q.W.); fyyypanmin@fjmu.edu.cn (M.P.); cqinyefj@fjmu.edu.cn (Q.-Y.C.); guilongxin@fjmu.edu.cn (L.-X.G.); 2 Key Laboratory of Fujian Province Universities on Ion Channel and Signal Transduction in Cardiovascular Diseases, Fujian Medical University, Fuzhou 350122, China; linechen@fjmu.edu.cn (Y.-C.L.); zhengsiyic@fjmu.edu.cn (S.-Y.Z.); 3 Department of Vascular Surgery, The First Affiliated Hospital, Fujian Medical University, Fuzhou 350005, China; 4 Department of Vascular Surgery, National Regional Medical Center, Binhai Campus of the First Affiliated Hospital, Fujian Medical University, Fuzhou 350212, China; 5 Department of Epidemiology and Health Statistics, School of Public Health, Fujian Medical University, Fuzhou 350122, China; 6 NHC Key Laboratory of Etiological Epidemiology of Chronic Diseases with High Incidence in Fujian-Taiwan Area (Co-Construction), Fujian Medical University, Fuzhou 350122, China

**Keywords:** abdominal aortic aneurysm, TRPC1, TRPM8, store-operated calcium entry, vascular smooth muscle cells

## Abstract

Abdominal aortic aneurysm (AAA) is a life-threatening vascular disease characterized by vascular smooth muscle cell (VSMC) dysfunction and disrupted calcium homeostasis. While transient receptor potential canonical 6 (TRPC6) and transient receptor potential canonical 1 (TRPC1) are known to mediate receptor-operated calcium entry (ROCE) and store-operated calcium entry (SOCE), respectively, the specific contributions of SOCE and ROCE to AAA pathogenesis, and the regulatory interaction between transient receptor potential melastatin 8 (TRPM8) and TRPC1 remain unexplored. In this study, we analyzed human AAA tissues, a papain-induced mouse model, and angiotensin II (Ang II)-treated human aortic smooth muscle cells using histology, wire myography, calcium imaging, and patch-clamp electrophysiology. We observed significant upregulation of TRPM8, TRPC1, and TRPC6 in both human and experimental AAA, with TRPC1 identified as a key mediator of SOCE under pathological conditions. Pharmacological activation of TRPM8 by menthol attenuated TRPC1-mediated SOCE and associated vasoconstriction, effects that were partially reversed by the TRPM8 antagonist A-2. In Ang II-treated cells, TRPM8 activation reduced SOCE and store-operated calcium currents (I_SOCC_), effects that were largely abolished by TRPC1 knockdown. These findings suggest that TRPM8 may limit excessive calcium ion (Ca^2+^) influx and vascular remodeling in AAA, pointing to a potential endogenous mechanism to counteract maladaptive calcium signaling in AAA progression.

## 1. Introduction

Abdominal aortic aneurysm (AAA) is a critical vascular condition defined by a focal aortic dilation exceeding 50% of the normal diameter [1]. Despite the rising prevalence driven by aging populations, effective pharmacological interventions to arrest aneurysm expansion remain elusive. Current management relies heavily on surveillance for small aneurysms and surgical repair for those at high risk of rupture, leaving a significant therapeutic gap [2,3,4]. Given that rupture is associated with mortality rates exceeding 80%, identifying the molecular drivers of AAA progression is essential for developing non-surgical therapies.

The pathogenesis of AAA is complex, involving extracellular matrix (ECM) degradation, inflammation, and, critically, the dysfunction of vascular smooth muscle cell (VSMC) [5]. Under pathological stress, VSMCs undergo phenotypic switching and apoptosis, processes tightly governed by intracellular calcium ([Ca^2+^]_i_) homeostasis. Sustained [Ca^2+^]_i_ elevation acts as a primary trigger for aberrant vasoconstriction and maladaptive vascular remodeling [5].

In VSMC, specifically during pathological remodeling, this calcium influx is predominantly mediated by Store-Operated Calcium Entry (SOCE), a mechanism activated by the depletion of sarcoplasmic reticulum stores [6]. While receptor-operated calcium channels, including the transient receptor potential canonical 6 (TRPC6), have been implicated in calcium dysregulation across various vascular pathologies, their role in AAA appears distinct [7,8]. For instance, in the widely used Angiotensin II (Ang II)-induced mouse model of AAA, transcriptomic analysis revealed a dynamic expression pattern of *TRPC6*: its messenger RNA (mRNA) was downregulated in the early disease stage but recovered later. Despite this alteration, pharmacological inhibition of TRPC6 fails to alter AAA progression in vivo [9]. In stark contrast, genetic deficiency of endothelial transient receptor potential vanilloid subtype 4 (TRPV4) channels—which are upregulated in early-stage AAA—demonstrates a clear protective effect, ameliorating experimental AAA [9]. This underscores a critical principle: not all calcium channels with altered expression are pathogenic drivers in AAA. Within the vasculature, the transient receptor potential canonical 1 (TRPC1) channel serves as a core component of Store-Operated Ca^2+^ Channels (SOCC) [6]. Elevated TRPC1 activity and enhanced SOCE have been implicated in various cardiovascular pathologies, including hypertension and aortic dissection, where they drive VSMC proliferation and maladaptive remodeling [10,11,12]. In AAA, we hypothesize: early upregulation of TRPC1 likely mediates excessive calcium ion (Ca^2+^) influx, thereby promoting VSMC phenotypic switching, ECM degradation, and proliferative remodeling, consistent with patterns observed in other proliferative vascular diseases [13].

In contrast to the pro-remodeling effects of TRPC1, the transient receptor potential melastatin 8 (TRPM8)—classically known as a cold sensor—appears to exert a protective vascular effect [14,15]. Activation of TRPM8 by agonists such as menthol induces vasorelaxation and attenuates hypertensive responses by modulating Ras homolog gene family member A (RhoA)/Rho-associated coiled-coil containing protein kinase (ROCK) signaling and oxidative stress [16,17]. In pulmonary hypertension, TRPM8 is downregulated, and the vasorelaxation effect is inhibited, leading to the strongest pulmonary artery contraction and remodeling. Our previous work in pulmonary hypertension confirms that TRPM8 activation functions as a negative regulator of SOCE, thereby reducing [Ca^2+^]_i_ levels and promoting vasorelaxation in pulmonary arteries [18,19,20]. However, it remains entirely unknown whether TRPM8 contributes to vascular tone regulation in AAA, or whether this channel can functionally antagonize TRPC1-mediated SOCE to counteract calcium influx and aberrant vasoconstriction in aneurysmal pathology.

Here, we utilized human AAA tissues, a papain-induced mouse model, and Ang II-stimulated human aortic smooth muscle cell (HASMC) to characterize the interplay between TRPM8 and TRPC1. By combining wire myography, calcium imaging, and patch-clamp electrophysiology, we investigated the mechanistic interplay between TRPM8 and TRPC1, aiming to establish TRPM8 as a novel therapeutic target for AAA intervention.

## 2. Materials and Methods

### 2.1. Human Samples

Aortic tissues were collected during open surgical AAA repair. Aneurysmal segments were obtained from all 6 patients, while adjacent non-dilated segments were taken from the non-branching aortic wall at the proximal or distal neck of the aneurysm in 4 of these patients (n = 4). All specimens were immediately snap-frozen at −80 °C. Ethics approval: First Affiliated Hospital of Fujian Medical University (approval number: [2021]169); all participants gave informed consent. Patients were eligible for inclusion if they had infrarenal AAA confirmed by computed tomography angiography or magnetic resonance imaging, with a maximum aneurysm diameter of ≥3.0 cm, and were scheduled for open or endovascular repair. Patients were excluded if they had autoimmune disease, active infection, malignancy, or pregnancy.

### 2.2. Murine AAA Models

All procedures were approved by the Fujian Medical University Institutional Animal Care and Use Committee (IACUC, approval number: FJMU2023-Y-0618) and reported following Animal Research: Reporting of In Vivo Experiments (ARRIVE 2.0). The experimental unit was the individual mouse. Mice were housed at 22–24 °C, 50–70% humidity, 12 h light/dark with autoclaved chow and acidified water ad libitum. Same-group caging was employed (i.e., mice within a single cage received the same treatment) to prevent cross-contamination. Cage positions on the rack were randomized to control for potential environmental gradients. A total of 14 male C57BL/6 mice (8–10 weeks old, 20–25 g) were used in this study. Animals were randomly assigned to the control group (CON, n = 7) and the papain-induced AAA model group (AAA-Papain, n = 7). All animals were naive and had no prior surgical history. No formal statistical sample size calculation was performed prior to the study. The sample size (n = 7 per group) was determined based on experience from our previous similar studies [21] and in accordance with the 3R principles (Replacement, Reduction, Refinement) of animal ethics, aiming to ensure detection of biologically meaningful differences between groups while minimizing the number of animals used.

### 2.3. Papain-Induced C57BL/6 Model

Male C57BL/6 mice (8–10 weeks old) were anaesthetised with pentobarbital (45 mg kg^−1^ i.p.). A cotton pledget soaked in papain solution (40 mg mL^−1^, 80 µL, P4762, Sigma-Aldrich, St. Louis, MO, USA) was placed around the infrarenal aorta for 20 min; control animals received an identical pledget soaked in saline only and applied in the same manner, as previously described [21]. For post-operative analgesia, animals received buprenorphine (0.1 mg kg^−1^) plus meloxicam (1 mg kg^−1^) for 3 days. Animals were randomly allocated to experimental groups using a random number table. All surgical procedures were performed by the same operator at a fixed time each day to minimize diurnal variation. Exclusion criteria included unexpected death during surgery, severe postoperative infection, wound dehiscence, or congenital aortic abnormalities detected by ultrasound. No animals were excluded from analysis in this study.

### 2.4. Endpoint and Outcome Measures

Mice were euthanised after 14 days with isoflurane and cervical dislocation. Aortas were perfused with ice-cold phosphate-buffered saline (PBS) and dissected for analysis.

Primary outcome measures assessed at sacrifice included the maximum abdominal aortic diameter, quantified by ultrasound at day 14 and confirmed by macroscopic measurement. AAA was defined as a >50% increase in maximum aortic diameter compared to the adjacent normal aortic segment. Comprehensive histological evaluation of aortic wall structure included wall thickness by hematoxylin and eosin (HE) staining, collagen deposition by Masson’s trichrome staining, and elastin integrity by Elastica van Gieson (EVG) staining. Secondary outcome measures included matrix metalloproteinase (MMP) expression levels (MMP2 and MMP9).

### 2.5. Ultrasound

Maximum abdominal aortic diameter was measured in anesthetized mice (2% isoflurane, R510-22-10, RWD, Shenzhen, China) using high-resolution ultrasound (FUJIFILM Sonosite HSL25, Bothell, WA, USA) in a blinded, randomised fashion. All ultrasound measurements were performed by an independent investigator who was blinded to the group allocation. Image analysis and diameter measurements were conducted using ImageJ (ver. 1.53a) under blinded conditions to ensure objective assessment of outcomes.

### 2.6. Culture and Treatment

Immortalised human aortic smooth-muscle cell (IHASMC, CTCC-001-0577, Meisen CTCC, Hangzhou, China) were cultured in high-glucose Dulbecco’s Modified Eagle Medium (DMEM, 11995-065, Gibco, Thermo, Waltham, MA, USA) supplemented with 10% (*v*/*v*) fetal bovine serum (FBS, 16000-044, Gibco, Thermo, Waltham, MA, USA) and 1% penicillin-streptomycin (15140-122, Gibco, Thermo, Waltham, MA, USA) at 37 °C, 5% CO_2_. For the experiments conducted, cells were cultivated until they reached 70–80% confluence. Cells underwent a pre-treatment with Ang II (4474-91-3, MCE, Shanghai, China) for a duration of 24–48 h. The vehicle control groups were administered an equivalent volume of dimethyl sulfoxide (DMSO, D2650, Sigma-Aldrich, St. Louis, MO, USA) at a concentration of 0.1% (*v*/*v*).

The following small interfering RNA (siRNA) targeting TRPC1 (siTRPC1) sequences were used: sense, 5′-GGGUCCAUUACAGAUUUCATT-3′; antisense, 5′-UGAAAUCUGUAAUGGACCCTT-3′. Small interfering RNA negative control (siNC) served as the negative control. All siRNAs were dissolved in nuclease-free water to a stock concentration of 20 μM and transfected at a final concentration of 50 nM using EntransterTM-R4000 (4000-4, Engreen Biosystem, Beijing, China) according to the manufacturer’s protocol.

### 2.7. Real-Time Quantitative PCR (RT-qPCR)

Total RNA was isolated from AAA tissues or cultured cells utilizing TRIzol reagent (15596018CN, Thermo, Waltham, MO, USA). The synthesis of complementary DNA (cDNA) was conducted using the PrimeScript RT reagent kit (RR037A, Takara, Kusatsu, Japan), adhering to the manufacturer’s guidelines. RT-qPCR was performed on a CFX96 Touch system (Bio-Rad, Hercules, CA, USA) employing TransStart Green qPCR SuperMix (RR42A, Takara, Kusatsu, Japan). The cycling parameters consisted of an initial denaturation step at 95 °C for 10 min, followed by 40 amplification cycles at 95 °C for 15 s and 60 °C for 60 s. To ensure the specificity of the amplification, a melt-curve analysis was included with the following conditions: 95 °C for 15 s, 60 °C for 60 s, and a final step at 95 °C for 15 s. Gene expression levels were normalized against Glyceraldehyde-3-Phosphate Dehydrogenase (GAPDH) and quantified using the 2^−ΔΔCt^ method. Each assay was conducted in triplicate, and all experimental procedures were repeated three times. The sequences of the primers utilized are provided in Table S1.

### 2.8. Western Blot

Protein lysates were obtained using radioimmunoprecipitation assay (RIPA) buffer (P0013B, Biosystem, Beijing, China) that was enriched with a cocktail of protease and phosphatase inhibitors (HY-K0021, MCE, Shanghai, China). Following sonication and centrifugation at 13,000× *g* for 20 min at 4 °C, the protein concentration was quantified utilizing the Pierce bicinchoninic acid (BCA) kit (P0011, Biosystem, Beijing, China). Subsequently, equivalent protein quantities (20–30 µg per lane) were subjected to separation via 8–12% sodium dodecyl sulfate-polyacrylamide gel electrophoresis (SDS-PAGE) gels (AR0138, BOSTER, Wuhan, China) before being transferred to polyvinylidene difluoride (PVDF) membranes (Millipore, Burlington, VT, USA). Membranes were blocked with 5% non-fat milk in Tris-buffered saline with Tween 20 (TBST) for 1 h at room temperature and incubated overnight at 4 °C with the following primary antibodies: TRPC1 (1:1000, 19482-1-AP, Proteintech, Wuhan, China), TRPM8 (1:2000, DF7966, Affinity, Changzhou, China), MMP2 (1:1000, AF5330, Affinity, Changzhou, China), MMP9 (1:1000, AF5228, Affinity, Changzhou, China), and β-actin (1:10,000, BS6007M, Bioworld, St. Louis Park, MN, USA). Subsequent to performing three washes with TBST, the membranes were allowed to incubate at room temperature for one hour with horseradish peroxidase (HRP)-conjugated secondary antibodies at a dilution of 1:5000 (#7074 or #7076, Cell Signaling, Danvers, MA, USA). Immunoreactive bands were visualised with enhanced chemiluminescence (ECL) substrate (Thermo, Waltham, MO, USA) and quantified using ChemIImager 5500 v2.03 software.

### 2.9. Immunohistochemistry (IHC)

For the IHC assay, aorta sections embedded in paraffin were placed in sodium citrate for microwave antigen retrieval (5–7 min) and per meabilized with 0.05% Triton-PBS for 30 min at room temperature. Sections were blocked with 5% bovine serum albumin (BSA) for 45 min, incubated overnight at 4 °C with primary antibodies: TRPC1 (1:200, 19482-1-AP, Proteintech, Wuhan, China), TRPM8 (1:200, DF7966, Affinity, Changzhou, China), MMP2 (1:200, AF5330, Affinity, Changzhou, China), MMP9 (1:200, AF5228, Affinity, Changzhou, China), and then with HRP-conjugated secondary antibodies (K4063, Dako, Glostrup, Denmark) for 1 h at room temperature. The staining procedure was conducted utilizing a 3,3′-Diaminobenzidine (DAB) substrate, followed by counterstaining with hematoxylin. Images were obtained using an Olympus BX43 (Tokyo, Japan) upright microscope.

### 2.10. Calcium Imaging

HASMC were loaded with Fluo-3 AM (2 μM, S1056, Biosystem, Beijing, China) for 30 min at 37 °C. Calcium influx was monitored by confocal microscopy (C2; Nikon, Tokyo, Japan) after stimulation with Menthol (10^−3.5^ M, m2772, Sigma-Aldrich, St. Louis, MO, USA) or TRPM8 antagonist 2 (A-2, 10^−7^ μM, HY-112430, MCE, Shanghai, China). Changes in fluorescence intensity were quantified with ImageJ v1.53a. After analysis by the FeliX 32 software, the [Ca^2+^]_i_ value was calculated using the following formula:

[Ca^2+^]_i_ = Kd × (F − F_bg_)/(F_max_ − F), where Kd = 1100 nM for the Ca^2+^ indicator used. F is the measured fluorescence intensity, F_bg_ is the background (minimum) fluorescence, and F_max_ is the maximum fluorescence obtained under saturating Ca^2+^ conditions.

### 2.11. Histology

Aortic sections were preserved using 4% paraformaldehyde, subsequently dehydrated, and then embedded in paraffin. Five-micrometre sections were deparaffinised, rehydrated and stained with HE kit (ab245880, Abcam, Cambridge, UK), EVG kit (ab150667, Abcam, Cambridge, UK) and Masson’s trichrome kit (HT15-1KT, Sigma-Aldrich, St. Louis, MO, USA). Slides were scanned on an EasyScan digital slide scanner (Motic EasyScan Pro 6, Motic, Xiamen, China). Inflammatory and fibrotic areas were quantified at ×100 and ×400 magnification using ImageJ (ver. 1.53a) and expressed as a percentage of the total field [21].

### 2.12. Vascular Functional Studies by Wire Myography

Abdominal aortas were isolated from mice in the CON and AAA-Papain groups. After euthanasia, the abdominal aorta was rapidly excised and placed in ice-cold, oxygenated modified Krebs solution containing (in mM): 118.3 NaCl, 1.2 MgSO_4_, 1.2 KH_2_PO_4_, 25 NaHCO_3_, 0.016 EDTA, 10.0 Glucose, and 2.0 CaCl_2_. Perivascular fat and connective tissue were carefully removed under a stereomicroscope. For experiments performed in endothelium-denuded rings, the endothelium was mechanically disrupted by gently rubbing the luminal surface with a fine hair, as described previously, and rings were cut into segments of approximately 1.8–2.0 mm in length.

Aortic rings were mounted on a wire myograph (Model 620M; Danish Myo Technology A/S, Aarhus, Denmark) using two stainless-steel wires (40 μm diameter). Vessels were bathed in Krebs solution gassed with 95% O_2_ and 5% CO_2_ and maintained at 37 °C (pH 7.4). Isometric tension was recorded and analyzed using LabChart 8 (AD Instruments, Bella Vista, Australia). After a 30 min equilibration period, rings were normalized to the optimal internal circumference using the normalization procedure with a target transmural pressure of 100 mmHg, followed by an additional 60 min equilibration with regular buffer changes.

To assess vascular smooth muscle viability and establish a reference contraction, rings were stimulated with 60 mM potassium chloride (KCl, A100395, Diamond, Shanghai, China). The 60 mM KCl solution was prepared by equimolar replacement of NaCl in the normal Krebs solution to maintain isotonicity (i.e., NaCl reduced from 120 mM to 64.7 mM, with all other components unchanged). After washout and re-stabilization, the rings were pre-constricted with the α-adrenergic agonist phenylephrine (PE, 10 μM, P0398, Tokyo Chemical Industry, Tokyo, Japan). For TRPM8-dependent vasorelaxation experiments, rings were preconstricted with PE (10 μM) and then exposed to cumulative concentrations of menthol (10^−7^–10^−3.5^ M) to generate concentration–response curves. Based on these curves, a single concentration of menthol (10^−3.5^ M) was used in subsequent assays. Where indicated, tissues were pretreated with A-2 (10^−7^ M) before menthol application to assess specificity of TRPM8-mediated relaxation.

SOCE-mediated contraction was assessed using cyclopiazonic acid (CPA, 10 μM, C1530, Sigma-Aldrich, St. Louis, MO, USA) under Ca^2+^-free conditions, followed by extracellular Ca^2+^ add-back. Briefly, rings were incubated in nominally Ca^2+^-free Krebs solution and stimulated with CPA to deplete sarcoplasmic reticulum Ca^2+^ stores; extracellular Ca^2+^ was then reintroduced to evoke SOCE-dependent contraction. To determine the effect of TRPM8 activation on SOCE, rings were preincubated with menthol (10^−3.5^ M) prior to CPA stimulation, and in some experiments, A-2 (10^−7^ M) was applied together with menthol to test whether menthol-dependent inhibition of CPA-induced contraction was TRPM8 mediated. Contractile force was normalized to vessel wet weight for group comparisons, and relaxation responses were expressed relative to the level of PE-induced preconstriction, as indicated.

### 2.13. Whole-Cell Patch-Clamp Recording

HASMC were used to investigate the electrophysiological interaction between TRPM8 activation and TRPC1-mediated SOCE. HASMC were divided into Vehicle and Ang II-treated groups to model AAA-like signaling in vitro. Cells were plated onto glass coverslips and used for recordings under an inverted microscope at room temperature.

Whole-cell patch-clamp recordings were performed using standard techniques. Patch pipettes were pulled from borosilicate glass and fire-polished to obtain an appropriate tip resistance (typically 3–6 MΩ when filled with pipette solution). After forming a gigaseal, the whole-cell configuration was established and membrane capacitance was compensated; series resistance was monitored throughout the experiment, and recordings were excluded if series resistance changed substantially during acquisition. Currents were acquired with an Axon 200B patch-clamp amplifier and a Digidata 1550A digitizer (both from Axon Instruments, Foster City, CA, USA) for offline analysis. Current density was calculated by normalizing current amplitude to membrane capacitance (pA/pF).

To evoke Store-Operated Calcium Currents (I_SOCC_), cells were voltage-clamped at a holding potential of −70 mV. CPA (10^−5^ M) was applied to deplete intracellular Ca^2+^ stores, resulting in a gradual development of inward whole-cell currents. To confirm the SOCE/I_SOCC_ identity, lanthanum ion (La^3+^, 10^−3^ M), a non-selective SOCE blocker, was subsequently applied to inhibit CPA-activated currents. Current–voltage (I–V) relationships were obtained using a voltage-step protocol spanning −70 mV to +70 mV, and representative currents and I–V curves were constructed for Vehicle and Ang II-treated groups.

To determine whether TRPM8 activation modulates CPA-induced I_SOCC_, CPA-induced currents were first allowed to stabilize, after which menthol (10^−3.5^ M) was applied and currents were re-measured using the same voltage protocol (−70 mV to +70 mV). Finally, La^3+^ (10^−3^ M) was applied in the continued presence of CPA (and menthol where applicable) to abolish residual current. For group comparisons, current density at +70 mV and the corresponding I–V relationships were quantified, and the extent of menthol- and La^3+^-induced inhibition was calculated relative to the CPA-activated baseline.

All electrophysiological experiments were conducted at room temperature (20–25 °C) using an Axon 200B patch-clamp amplifier for recording. Experimental parameters were controlled, data were acquired, and stimulation was delivered using Clampex 10.5 software (Axon Instruments, Foster City, CA, USA).

### 2.14. Statistical Analysis

Data are expressed as mean ± standard deviation (SD). All statistical analyses were performed based on biological replicates (n represents the number of animals per group). Prior to parametric tests, normality of data distribution was assessed using the Shapiro–Wilk test, and homogeneity of variances was assessed using Levene’s test. All data met the assumptions of normal distribution and homogeneity of variance. The assessment of differences between two groups was conducted utilizing two-tailed unpaired Student’s *t*-tests. For multiple comparisons, data were analyzed using one-way or two-way analysis of variance (ANOVA), followed by Tukey’s post hoc test. *p* < 0.05 was deemed statistically significant. Graphical representations were created with GraphPad Prism (version 10.4.0, GraphPad Software) and subsequently compiled in Adobe Illustrator 2024 (Adobe Systems). Data analysis was also performed using software including Image-Pro Plus 6.0, pClamp 10.5, NIS-Elements AR 4.50.00, and ImageJ 1.53a.

## 3. Results

### 3.1. Upregulation of TRPC1/TRPM8 Coincides with Matrix Degradation and Aortic Wall Remodeling in AAA Patients

**Figure 1:**
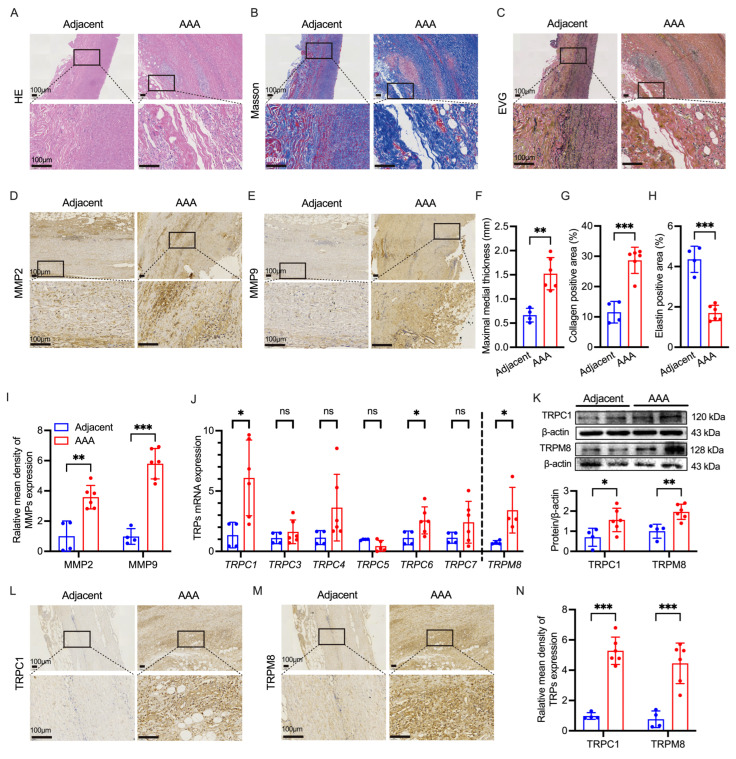
Histological characteristics and TRP channel expression in human AAA tissues. (**A**–**C**) Representative images of aortic tissues from the Adjacent and AAA groups. Sections were stained with HE (**A**), Masson’s trichrome (**B**), and EVG (**C**) to visualize general morphology, collagen deposition, and elastic fibers, respectively. (**D**,**E**) Representative immunohistochemical staining for MMP2 (**D**) and MMP9 (**E**) in the aortic wall. (**F**–**H**) Quantitative analysis corresponding to the histological staining: wall remodeling (**F**), collagen area (**G**), and elastin degradation score (**H**). (**I**) Quantification of MMP2- and MMP9-positive areas. (**J**) RT-qPCR screening of TRP channels mRNA expression in human AAA tissues compared to the Adjacent group. (**K**) Western blot analysis of TRPC1 and TRPM8 protein levels. Top: representative blots; Bottom: densitometric analysis. (**L**,**M**) Representative immunohistochemical staining showing the localization of TRPC1 (**L**) and TRPM8 (**M**) in aortic tissues. (**N**) Quantitative analysis of TRPC1 and TRPM8 expression levels. Data are presented as mean ± SD. ns *p* > 0.05, * *p* < 0.05, ** *p* < 0.01, *** *p* < 0.001 vs. Adjacent (Adjacent: n = 4; AAA: n = 6). Scale bar represents 100 μm. Original images of (**K**) can be found in Supplementary Materials.

Histological analysis was performed to characterize structural alterations in AAA tissues compared to non-dilated adjacent segments (Adjacent group). HE staining revealed extensive remodeling in the AAA group, characterized by significant aortic wall thickening and inflammatory infiltration compared to the intact architecture of the adjacent vessels (Figure 1A,F). Evaluation of the ECM confirmed loss of vascular integrity. Masson’s trichrome staining indicated pronounced collagen deposition and fibrosis in the aneurysmal wall (Figure 1B,G). Concurrently, EVG staining demonstrated severe fragmentation and depletion of elastic fibers, contrasting with the orderly, wavy elastic lamellae observed in the Adjacent group (Figure 1C,H).

Consistent with these elastolytic changes, immunohistochemical analysis showed marked upregulation of gelatinases within the vessel wall. Expression levels of both MMP2 (Figure 1D,I) and MMP9 (Figure 1E,I) were significantly elevated in AAA tissues compared to the Adjacent group, suggesting that proteolytic hyperactivity contributes to the observed ECM degradation.

To explore the ionic mechanisms underlying this pathology, we screened for the expression of transient receptor potential (TRP) channels. RT-qPCR analysis revealed a distinct expression profile in AAA tissues: mRNA levels of *TRPC1*, *TRPC6*, and *TRPM8* were significantly upregulated (Figure 1J). No significant differences were observed for *TRPC3*, *TRPC4*, *TRPC5*, or *TRPC7* mRNA levels. Based on these findings, we selected TRPC1 and TRPM8 for protein-level validation. Western blot analysis confirmed that TRPC1 and TRPM8 protein levels were significantly increased in AAA samples (Figure 1K). Immunohistochemical staining further corroborated these results, showing intense localization of TRPC1 (Figure 1L,N) and TRPM8 (Figure 1M,N) in the aneurysmal tissues. These data indicate that the upregulation of TRPC1 and TRPM8 channels is a prominent molecular feature of human AAA.

### 3.2. Pathological Features and Altered TRPC1/TRPM8 Expression in the Papain-Induced AAA Mouse Model

**Figure 2:**
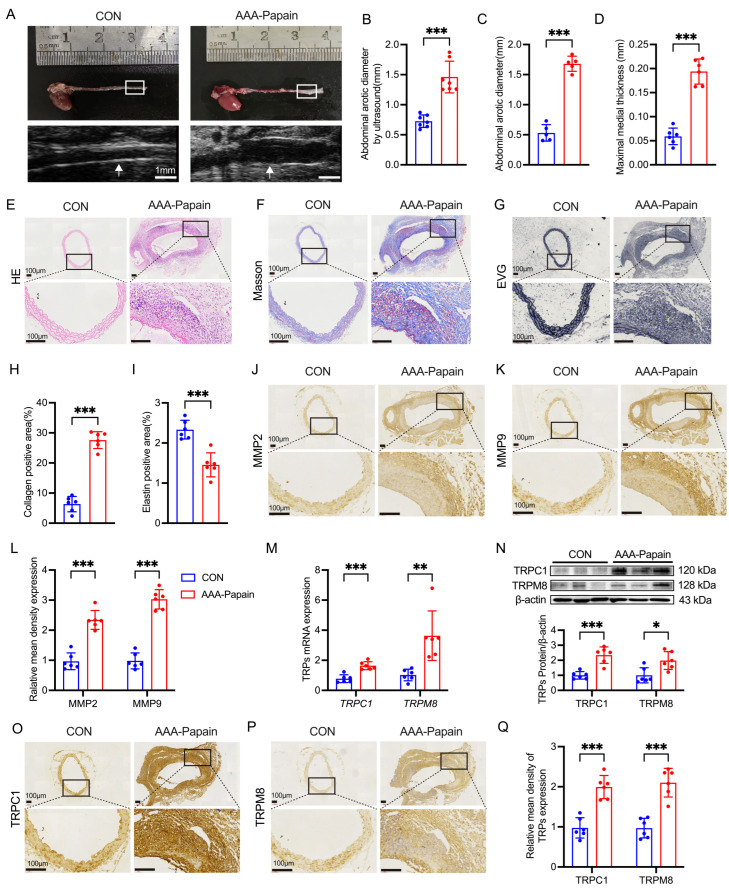
Characterization of the papain-induced AAA mouse model and TRP channel expression. (**A**) Representative images of gross aortic morphology and Doppler ultrasound scans from the CON and AAA-Papain groups 14 days post-surgery. White boxes (gross) and arrows (ultrasound) indicate the aneurysm (AAA-Papain) and the corresponding aorta (CON). (**B**,**C**) Quantitative analysis of the maximum abdominal aortic diameter measured by ultrasound (n = 7 per group) (**B**) and macroscopic examination (n = 5 per group) (**C**). (**E**–**G**) Representative histological images of aortic sections stained with HE (**E**), Masson’s trichrome (**F**), and EVG (**G**) to assess wall architecture, collagen deposition, and elastic fiber integrity (n = 6 per group). (**D**,**H**,**I**) Quantitative analysis corresponding to the histological features: medial thickness (**D**), collagen-positive area (**H**), and elastin degradation score (n = 6 per group) (**I**). (**J**,**K**) Representative immunohistochemical staining for MMP2 (**J**) and MMP9 (**K**) in the murine aortic wall. (**L**) Quantification of MMP2- and MMP9-positive areas. (**M**) RT-qPCR analysis of *Trpc1* and *Trpm8* mRNA expression levels in aortic tissues (n = 6 per group). (**N**) Western blot analysis of TRPC1 and TRPM8 protein abundance. Top: representative blots; Bottom: densitometric analysis. (**O**,**P**) Representative immunohistochemical staining showing the localization of TRPC1 (**O**) and TRPM8 (**P**) in the aortic tissues. (**Q**) Quantitative analysis of TRPC1 and TRPM8 expression (n = 6 per group). Data are presented as mean ± SD. * *p* < 0.05, ** *p* < 0.01, *** *p* < 0.001 vs. CON. Scale bar represents 100 μm. Original images of (**N**) can be found in Supplementary Materials.

To evaluate the efficacy of the papain-induced model (AAA-Papain), abdominal aortic diameters were assessed via Doppler ultrasound and gross anatomical examination 14 days post-surgery. Ultrasound imaging revealed a significant expansion in the aortic diameter of the AAA-Papain group compared to the CON group (Figure 2A,B). Consistent with the sonographic findings, macroscopic measurements of the infrarenal aorta confirmed that the vessel diameter in the AAA-Papain group was significantly increased relative to the CON group (Figure 2A,C). The aneurysmal dilation exceeded a 50% increase compared to the normal vessel segment, indicating the successful establishment of the murine AAA-Papain model.

Histological staining was performed to characterize the structural alterations induced by papain. HE staining demonstrated that while the CON group maintained a distinct and intact vessel architecture with a thin wall, the AAA-Papain group exhibited marked medial thickening and hyperplasia (Figure 2D,E). Masson’s trichrome staining revealed a disorganized collagen network with significantly increased collagen deposition in the AAA-Papain group compared to the CON group (Figure 2F,H). Furthermore, EVG staining indicated a substantial reduction and fragmentation of elastic fibers in the aneurysmal wall (Figure 2G,I), reflecting severe ECM degradation.

To further investigate the proteolytic environment within the lesion, the expression of MMPs was evaluated by IHC. Compared to the baseline expression observed in the CON group, the AAA-Papain group showed significantly elevated immunoreactivity for both MMP2 (Figure 2J,L) and MMP9 (Figure 2K,L), confirming enhanced matrix remodeling activity in the model.

We next examined whether the TRP channel alterations observed in human tissues were recapitulated in the murine model. RT-qPCR analysis demonstrated that the mRNA levels of *Trpc1* and *Trpm8* were significantly upregulated in the AAA-Papain group compared to the CON group (Figure 2M). At the translational level, Western blot analysis confirmed a corresponding increase in TRPC1 and TRPM8 protein abundance in the AAA-Papain tissues (Figure 2N). These findings were further corroborated by immunohistochemical staining, which showed intense localization of TRPC1 and TRPM8 in the remodeled aortic wall of the AAA-Papain group (Figure 2O–Q). The expression patterns in the papain-induced mouse model were consistent with those observed in human AAA specimens.

### 3.3. Impaired Vascular Contractility in the AAA-Papain Mouse Model

**Figure 3:**
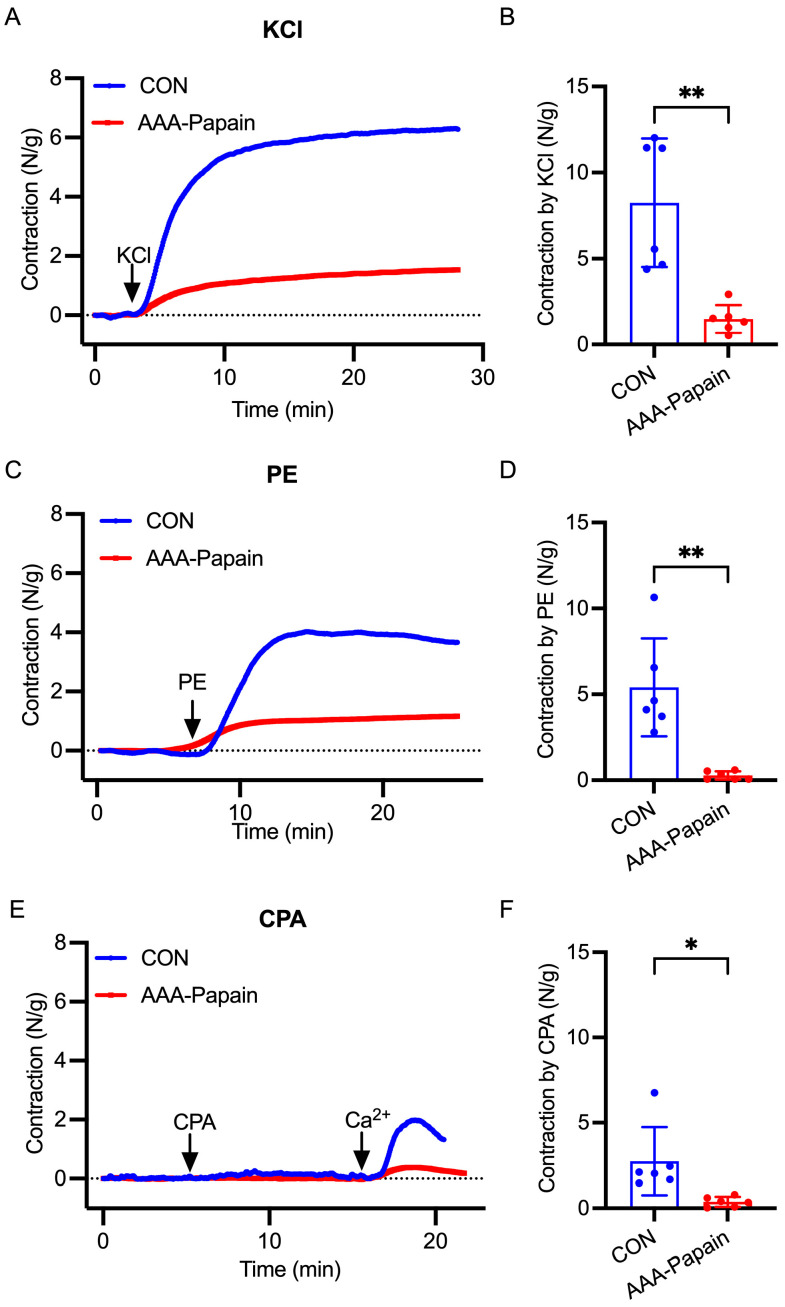
Assessment of vascular contractility in the AAA-Papain mouse model. (**A**) Representative isometric tension traces showing the contractile response to 60 mM KCl stimulation in aortic rings from the CON and AAA-Papain groups. (**B**) Quantitative analysis of KCl-induced contraction normalized to vessel weight. (**C**) Representative traces of contraction induced by the α-adrenergic agonist PE (10 μM). (**D**) Quantification of PE-induced contractile force. (**E**) Representative traces showing the contractile response to CPA (10 μM) in Ca^2+^-free solution followed by Ca^2+^ add-back, assessing SOCE-mediated contraction. The horizontal dashed line in (**A**,**C**,**E**) indicates the baseline tension. (**F**) Quantitative analysis of CPA-induced contraction. Data are presented as mean ± SD. * *p* < 0.05, ** *p* < 0.01, vs. CON (n = 6 per group).

Structural degeneration of the aortic wall, characterized by elastin fragmentation and collagen remodeling, compromises vascular integrity and VSMC function. To evaluate the functional consequences of these pathological changes, isometric tension measurements were performed using KCl, PE and CPA. When normalized to vessel weight, the contractile responses were consistently diminished in the AAA-Papain group compared to the CON group. Specifically, depolarization-induced contraction by KCl was significantly reduced in the AAA-Papain tissues (Figure 3A,B). Similarly, the contractile response to PE was markedly attenuated in the AAA-Papain group (Figure 3C,D). Furthermore, stimulation with CPA resulted in a significantly weaker contraction in the AAA-Papain group relative to the CON group (Figure 3E,F). These data indicate a generalized impairment of vascular contractility in the aneurysmal aorta, involving blunted responses to TRPC1-mediated SOCE activation.

### 3.4. Impact of TRPM8 Activation on TRPC1-Mediated SOCE in the AAA Mouse Model

To investigate the potential functional interaction between TRPM8 and TRPC1 channels, wire myography was employed to assess the effects of TRPM8 activation on SOCE and vascular contractility. The differential responses in the AAA-Papain model compared to the CON group were analyzed to elucidate the role of TRPM8 upregulation in disease pathology.

**Figure 4:**
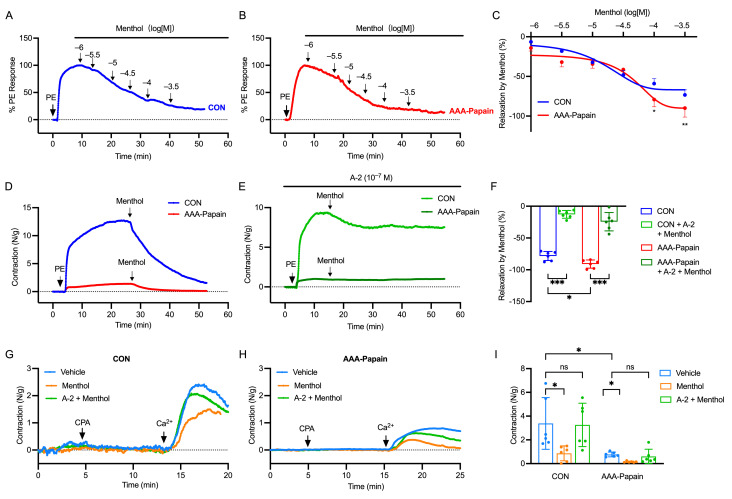
Activation of TRPM8 channels attenuates vasoconstriction and suppresses TRPC1-mediated SOCE in the AAA mouse model. (**A**,**B**) Representative traces showing the concentration-dependent relaxation induced by menthol in abdominal aortic rings precontracted with PE (10 μM) from the CON and AAA-Papain groups. (**C**) Concentration–response curves for menthol-induced relaxation. (**D**) Typical traces in the presence of a single dose of menthol (10^−3.5^ M) in PE-precontracted denuded abdominal aorta in the CON and AAA-Papain groups. (**E**) Typical traces of relaxation induced by a single dose of menthol (10^−3.5^ M) in the presence of A-2 (10^−7^ M) in PE-precontracted denuded abdominal aorta in the CON and AAA-Papain groups. (**F**) Quantitative analysis of the maximal relaxation response (E max) and the reversal of menthol-induced relaxation by A-2 pre-treatment. (**G**,**H**) Representative traces of CPA-induced contraction in the presence of vehicle, menthol (10^−3.5^ M), or menthol + A-2 (10^−7^ M) in the CON and AAA-Papain groups. The horizontal dashed line in (**A**,**B**,**D**,**E**,**G**,**H**) indicates the baseline tension. (**I**) Quantification of the CPA-induced contractile force under the indicated conditions. Data are presented as mean ± SD. ns *p* > 0.05, * *p* < 0.05, ** *p* < 0.01, *** *p* < 0.001 (n = 6 per group).

The vasoactive properties of the specific TRPM8 agonist, menthol, were evaluated in PE-preconstricted aortic rings. The concentration-response relationship was examined by cumulatively increasing menthol concentrations, such that each subsequent concentration was administered before the effect of the previous concentration had subsided (Figure 4A,B). Menthol administration induced a dose-dependent relaxation in both CON and AAA-Papain groups (Figure 4A,B). The maximal relaxation response was significantly greater in the AAA-Papain group compared to the CON group (Figure 4C), suggesting enhanced sensitivity to TRPM8 activation in the aneurysmal tissue. Based on these dose–response curves, a single concentration of menthol (10^−3.5^ M) was selected for subsequent experiments. To confirm the specificity of this response, the TRPM8 antagonist A-2 (10^−7^ M) was applied. Pre-treatment with A-2 significantly reversed the relaxant effect of menthol in both groups (Figure 4D,E), confirming that the observed vasodilation was mediated through the TRPM8 channel (Figure 4F). These results indicate that TRPM8 activation counteracts adrenergic vasoconstriction, an effect that is pronounced in the AAA-Papain model.

Given the observed impairment in contractility and the upregulation of TRPM8 in the AAA-Papain model, we further examined whether TRPM8 signaling modulates TRPC1-mediated SOCE. Aortic rings were pre-incubated with menthol (10^−3.5^ M) prior to induction of contraction with CPA, used here to induce SOCE. In the presence of menthol, the CPA-induced contractile response was significantly attenuated in both groups; however, the suppression was markedly more severe in the AAA-Papain group compared to the CON group (Figure 4G,H). This resulted in a significantly higher inhibition rate in the AAA-Papain tissues. The application of the TRPM8 antagonist A-2 (10^−7^ M) effectively reversed the inhibitory effect of menthol on CPA-induced contraction (Figure 4G–I). These findings suggest that TRPM8 activation negatively regulates TRPC1-mediated SOCE. The enhanced suppression observed in the AAA-Papain group correlates with the increased expression of TRPM8, implying that aberrant TRPM8 activity contributes to the reduced vascular contractility and altered calcium signaling characteristic of AAA.

### 3.5. Upregulation of TRPM8 and Its Regulation of SOCE in the Ang II-Induced HASMC Model

To explore a potential role of TRPC1 and TRPM8 channels in the phenotypic modulation of HASMCs by Ang II, we first established an in vitro model. Initial experiments showed that treatment with Ang II promoted HASMC proliferation in a manner dependent on both time and concentration (Figure S1). Based on these observations and existing literature, we selected 10^−7^ M Ang II for further investigation, as it induced a reproducible phenotypic shift.

**Figure 5:**
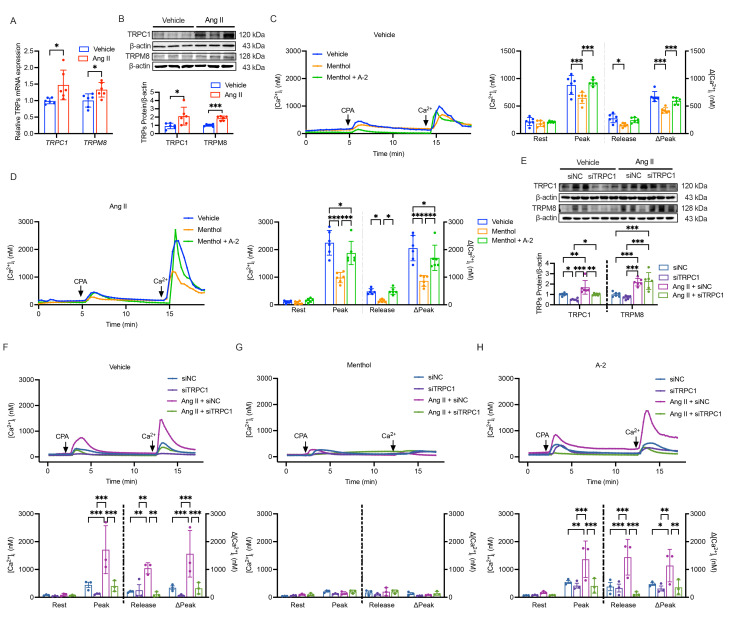
TRPM8 activation modulates Ang II-induced store-operated Ca^2+^ entry in HASMC. (**A**) Relative mRNA expression of *TRPC1* and *TRPM8* in HASMC treated with Vehicle or Ang II (10^−7^ M), determined by RT-qPCR (n = 6 per group). (**B**) Representative Western blot and densitometric analysis of TRPC1 and TRPM8 protein levels (n = 6 per group). (**C**) **Left**: Representative traces showing intracellular Ca^2+^ dynamics following store depletion with CPA (10^−5^ M) and subsequent extracellular Ca^2+^ restoration (2 mM) in Vehicle-treated groups, with vehicle, menthol (10^−3.5^ M), or menthol co-administered with the TRPM8 antagonist A-2 (10^−7^ M). **Right**: Quantification of resting Ca^2+^ levels, and the peak and net amplitude (ΔPeak) of CPA-induced Ca^2+^ release in Vehicle-treated groups, with or without menthol (10^−3.5^ M, n = 6 per group). (**D**) **Left**: Representative traces showing intracellular Ca^2+^ dynamics following store depletion with CPA (10^−5^ M) and subsequent extracellular Ca^2+^ restoration (2 mM) in Ang II-treated groups, with vehicle, menthol (10^−3.5^ M), or menthol co-administered with the TRPM8 antagonist A-2 (10^−7^ M). **Right**: Quantification of resting Ca^2+^ levels, and the peak and ΔPeak of CPA-induced Ca^2+^ release in Ang II-treated groups, with or without menthol (10^−3.5^ M, n = 6 per group). (**E**) Western blot analysis showing TRPC1 protein levels in HASMC transfected with siNC or siTRPC1, followed by vehicle or Ang II (10^−7^ M) treatment (n = 3 per group). (**F**) **Top**: Representative traces of Ca^2+^ dynamics in HASMC transfected with siNC or siTRPC1 and treated with vehicle or Ang II (10^−7^ M). **Bottom**: Quantitative analysis of SOCE, calculated as the peak Ca^2+^ influx (ΔPeak) after Ca^2+^ restoration (n = 3 per group). (**G**) **Top**: Representative traces showing the effect of menthol (10^−3.5^ M) on Ca^2+^ dynamics in HASMC transfected with siNC or siTRPC1 and treated with vehicle or Ang II (10^−7^ M). **Bottom**: Quantitative analysis of SOCE (ΔPeak) in the presence of menthol (n = 3 per group). (**H**) **Top**: Representative traces showing the effect of TRPM8 antagonist A-2 (10^−7^ M) on Ca^2+^ dynamics in HASMC transfected with siNC or siTRPC1 and treated with vehicle or Ang II (10^−7^ M). **Bottom**: Quantitative analysis of SOCE (ΔPeak) in the presence of A-2 (n = 3 per group). Data are presented as mean ± SD. * *p* < 0.05, ** *p* < 0.01, *** *p* < 0.001. Original images of (**B**,**E**) can be found in Supplementary Materials.

Consistent with our hypothesis, both the mRNA and protein levels of TRPC1 and TRPM8 were found to be elevated in HASMC treated with Ang II compared to vehicle-treated controls (Figure 5A,B). Based on these observations, we next investigated functional alterations in calcium handling. In cells subjected to store depletion with CPA, those pre-treated with Ang II exhibited a greater release of Ca^2+^ from intracellular stores and a subsequent larger influx of Ca^2+^ upon restoration of extracellular Ca^2+^, an index of SOCE (Figure 5C,D). This suggests that Ang II treatment may augment SOCE in these cells.

Interestingly, activation of the upregulated TRPM8 channel with its agonist menthol appeared to attenuate both the CPA-induced Ca^2+^ release and the SOCE-mediated influx, an effect that was particularly noticeable in Ang II-treated cells (Figure 5C,D). To determine whether menthol suppresses SOCE specifically via TRPM8 rather than through other calcium channels, we co-applied the TRPM8-selective antagonist A-2. A-2 largely prevented menthol-induced SOCE suppression, supporting an on-target, TRPM8-dependent mechanism rather than non-specific channel blockade.

To evaluate the contribution of TRPC1 to the observed changes, we utilized siRNA to reduce its expression. Western blot analysis confirmed effective knockdown of TRPC1 under both vehicle- and Ang II-treated conditions (Figure 5E and Figure S2A,B). In these TRPC1-knockdown cells, the enhanced SOCE typically observed following Ang II treatment was absent, suggesting that TRPC1 is required for mediating this specific aspect of the Ang II response (Figure 5F).

Furthermore, the inhibitory effect of menthol on SOCE appeared attenuated in cells where TRPC1 had been knocked down (Figure 5G). In TRPC1-deficient cells, the SOCE response was not further altered by the addition of the TRPM8 antagonist A-2 alone (Figure 5H). This may indicate that the regulatory influence of TRPM8 on SOCE involves, or is modulated by, the presence of functional TRPC1.

Together, these results are consistent with a model where Ang II upregulates both TRPC1 and TRPM8 in HASMC. While TRPC1 appears to be required for the augmented SOCE associated with the phenotypic change, TRPM8 activation conversely tempers this Ca^2+^ influx. The data further suggest that the inhibitory action of TRPM8 may be functionally linked to the presence of TRPC1, pointing to a potential interaction between these two channels in fine-tuning calcium signals during vascular smooth muscle cell adaptation.

### 3.6. TRPM8 Activation Modulates I_SOCC_ in Ang II-Induced HASMC

Given our observation that TRPM8 activation suppresses vascular contraction and Ca^2+^ influx in the AAA mouse model, we investigated the electrophysiological basis of this interaction using whole-cell patch-clamp techniques in HASMC. Specifically, we characterized the I_SOCC_ mediated by TRPC1 and assessed the impact of TRPM8 activation in Ang II-treated HASMC, which serve as an in vitro model of AAA.

**Figure 6:**
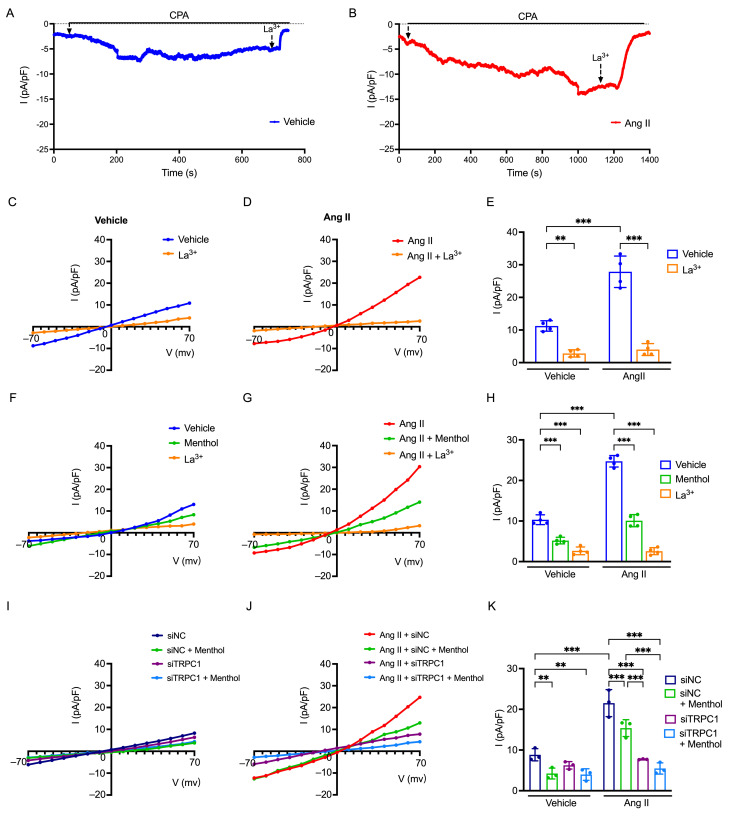
TRPM8 Activation Modulates I_SOCC_ in Ang II-Induced HASMC. (**A**,**B**) Time course of whole-cell currents activated by CPA (10^−5^ M) at a holding potential of −70 mV in Vehicle-treated HASMC and Ang II-treated HASMC. (**C**,**D**) I–V relationships showing the effects of CPA and La^3+^ in Vehicle (**C**) and Ang II groups (**D**). (**E**) Summary of current density (pA/pF) derived from whole-cell patch-clamp experiments involving CPA and La^3+^ in Vehicle and Ang II groups. Current densities (pA/pF) were quantified at +70 mV (derived from voltage steps from −70 mV to +70 mV, n = 4 per group). (**F**,**G**) I–V relationships showing the modulation by CPA, menthol and La^3+^ in Vehicle (**F**) and Ang II groups (**G**). (**H**) Summary of current density (pA/pF) in the presence of CPA, menthol, and La^3+^ in Vehicle and Ang II groups. Current densities (pA/pF) were quantified at +70 mV (derived from voltage steps from −70 mV to +70 mV, n = 4 per group). (**I**,**J**) I–V relationships showing the modulation by CPA and menthol in HASMC transfected with siNC or siTRPC1 and treated with vehicle (**I**) or Ang II group (**J**). (**K**) Summary of pA/pF in the presence of CPA and menthol in HASMC transfected with siNC or siTRPC1 and treated with vehicle or Ang II. Current densities (pA/pF) were quantified at +70 mV (derived from voltage steps from −70 mV to +70 mV, n = 3 per group). Data are presented as mean ± SD. ** *p* < 0.01, *** *p* < 0.001.

To record I_SOCC_, the membrane potential was held at −70 mV. Application of the SOCE inhibitor CPA (10^−5^ M) to deplete [Ca^2+^]_i_ stores induced a gradual activation of current in both Vehicle-treated and Ang II-treated HASMC. Subsequent application of the non-selective SOCE blocker La^3+^ (10^−3^ M) significantly inhibited these currents, confirming their identity as I_SOCC_ (Figure 6A,B). I–V relationships were generated using step protocols from −70 mV to +70 mV (Figure 6C,D). Analysis of the current density revealed that the CPA-induced I_SOCC_ was markedly elevated in Ang II-treated HASMC compared to Vehicle cells (*p* < 0.001; Figure 6E). Furthermore, the inhibitory effect of La^3+^ was significantly more pronounced in the Ang II-treated group (8.2-fold reduction) compared to the Vehicle group (4.4-fold reduction) at +70 mV (Figure 6E). These data indicate that I_SOCC_ density is upregulated in the Ang II-induced in vitro model, suggesting a functional increase in TRPC1-mediated channel activity.

We next tested the effect of TRPM8 activation on I_SOCC_. After establishing a stable CPA-induced current, menthol (10^−3.5^ M) was applied. To generate I–V curves, a voltage-step protocol ranging from −70 mV to +70 mV was applied repeatedly. Menthol administration resulted in a rapid reduction in current amplitude in both Vehicle and Ang II-treated groups. Analysis of the I–V curves of the I_SOCC_ and current density measured at a test potential of +70 mV (derived from these voltage steps) demonstrated that menthol partially inhibited I_SOCC_ in both groups (Figure 6F,G). Notably, the degree of inhibition was significantly greater in Ang II-treated HASMC (2.47-fold reduction) compared to Vehicle cells (1.98-fold reduction) (Figure 6H). Finally, the subsequent application of La^3+^ completely abolished the remaining current in both groups, reducing current density to near-baseline levels (Figure 6F–H).

TRPC1 knockdown markedly attenuated CPA-activated currents compared with siNC. The magnitude of this current suppression was comparable to that induced by La^3+^, which eliminated residual currents in both groups. This functional similarity between siRNA-mediated depletion and La^3+^ treatment is shown in Figure S2C–E. Moreover, the I–V relationships (Figure 6I,J) and current-density data (Figure 6K) show that, compared with the siNC group, the menthol-induced reduction of CPA-activated currents was significantly attenuated in TRPC1-knockdown cells, particularly following Ang II treatment.

Together, these patch-clamp data suggest that Ang II enhances the I_SOCC_, likely by up-regulating TRPC1 channel expression in smooth muscle cells. TRPM8 activation by menthol suppresses this current, and the attenuated response to menthol upon TRPC1 knockdown indicates that the menthol-sensitive component of the current is largely mediated by TRPC1-dependent I_SOCC_.

## 4. Discussion

In this study, we identified a paradoxical coexistence of elevated TRPC1, TRPC6, and TRPM8 expression with impaired vascular contractility in AAA. While upregulation of TRPC1 and TRPC6 was associated with enhanced SOCE and receptor-operated calcium entry (ROCE), respectively, pharmacological activation of TRPM8 substantially attenuated TRPC1-mediated SOCE, intracellular Ca^2+^ influx, and associated vasoconstriction, with this inhibitory effect being significantly potentiated under pathological conditions. These observations reveal a functional antagonism between TRPM8 and TRPC1 signaling within the aneurysmal wall, wherein TRPM8 activation counteracts the pro-contractile effects of TRPC1-mediated calcium influx, thereby contributing to the contractile dysfunction characteristic of advanced AAA.

### 4.1. Pathophysiological Remodeling in AAA and Model Validation

Aortic wall stability relies on the precise organization of the ECM. Consistent with established pathology [22,23], our histological analysis of human AAA tissues displayed characteristic maladaptive remodeling, including medial thickening, VSMC disarray, and extensive elastin fragmentation. While elastin provides necessary tensile strength and recoil, collagen maintains stiffness to prevent rupture when elastin is compromised [24]. This imbalance in the ECM likely reflects a maladaptive remodeling response that weakens aortic wall stability.

In line with these observations, we detected elevated expression of MMP2 and MMP9 in AAA tissues—enzymes associated with elastin and collagen breakdown [22,25,26]. To explore these mechanisms, we utilized the papain-induced mouse model, which effectively mimics the enzymatic degradation and medial degeneration observed clinically [21]. Our data suggest that this model provides a relevant platform for investigating the functional impairments associated with aneurysm development.

### 4.2. Altered Vascular Function and the TRP Channels

VSMC contractility plays a central role in regulating vascular tone and resisting hemodynamic stress. In AAA, a shift occurs from a contractile to a synthetic or senescent phenotype, contributing to progressive vessel dilation. Intracellular Ca^2+^ homeostasis, notably calcium influx via SOCE, is a key regulator of VSMC contractility [27,28]. TRPC1, acting within the SOCE machinery (typically alongside Orai1 and STIM1), supports the sustained Ca^2+^ influx required for contraction [29,30]. Our initial characterization of SOCE in AAA relied on the inhibitory effects of La^3+^, a broad-spectrum channel blocker. To strengthen the specificity of these findings, we employed siTRPC1 in subsequent experiments. The use of siTRPC1 produced a consistent reduction in store-operated inward currents, supporting the interpretation that TRPC1 contributes significantly to SOCE in this context. An apparent paradox exists in various cardiovascular conditions: despite increased TRPC1 expression, vascular contractility is often impaired—a pattern seen in diseases where dysregulated calcium handling leads to dysfunction rather than hypercontractility [29,31,32]. Such a disconnect between increased channel expression and reduced contractile function suggests additional regulatory mechanisms are involved.

Similarly, TRPC6, another member of the TRP channel family, exhibits upregulation in AAA and related vascular pathologies [33,34]. While TRPC6 facilitates Ca^2+^ influx and can contribute to vasoconstriction under normal conditions, its overexpression or upregulation in disease states appears to drive a phenotypic switch in VSMC from contractile to synthetic, leading to weakened contractile function and enhanced relaxation [33,35,36]. Potential mediating mechanisms include TRPC6-mediated Zn^2+^ influx that negatively regulates transforming growth factor beta (TGFβ)-induced contractile differentiation of VSMC [33], or plasma membrane potential-dependent coupling with phosphatase and tensin homolog (PTEN) that disrupts contractile protein expression and promotes dedifferentiation/proliferation [35]. In models of hypertension, pulmonary hypertension, and vascular remodeling, TRPC6 upregulation has been linked to reduced contractility, increased synthetic phenotype, and exacerbated vascular dysfunction, despite expectations of enhanced Ca^2+^ signaling [28,34,36].

The combined upregulation of TRPC1 and TRPC6 thus contributes to the observed impairment in vasoconstriction and augmentation of vasorelaxation, exacerbating vessel dilation in AAA. Given this, we hypothesized that TRPM8 upregulation may serve a modulatory role.

### 4.3. The Interplay Between TRPM8 and TRPC1 in AAA

Although best known as a cold-sensing channel, TRPM8’s vascular functions have garnered increasing interest. Our data confirm its abundant expression in the aorta [37]. Notably, activation of TRPM8 by menthol significantly attenuated TRPC1-mediated SOCE and associated vasoconstriction, effects that were more pronounced in AAA-affected vessels [16,18]. Although TRPM8 itself is permeable to Ca^2+^, its activation with menthol reduced PE-induced contraction and SOCE. To address whether these effects might arise from off-target actions of menthol, we utilized the selective TRPM8 antagonist A-2. Pretreatment with A-2 reversed the menthol-induced vasorelaxation and restored calcium signals and ionic currents that were diminished by menthol alone. These data, together with published evidence for menthol’s selectivity at TRPM8 [17,38,39], suggest that the inhibition of Ca^2+^ influx and tone is likely mediated specifically through TRPM8 activation. This aligns with observations in hypertension models, where TRPM8 activation promotes vasorelaxation through modulation of intracellular Ca^2+^ dynamics [16,19,40]. The precise mechanism underlying TRPM8’s inhibitory effect on TRPC1-mediated SOCE remains to be fully elucidated. Since both channels are regulated by phosphatidylinositol 4,5-bisphosphate (PIP2), TRPM8 activation may reduce local PIP2 availability or interfere with phospholipase C signaling, thereby limiting TRPC1 channel opening [41,42,43,44,45,46]. Electrophysiological evidence supports rapid suppression of sustained I_SOCC_ upon TRPM8 stimulation. This suggests that TRPM8 could act as a negative feedback “brake” on pathological Ca^2+^ influx. In AAA, TRPM8 upregulation might represent an adaptive response aimed at counteracting excessive Ca^2+^ influx.

Beyond direct calcium handling, TRPM8 may offer broader vascular protection. Emerging evidence indicates that TRPM8 can modulate inflammation and oxidative stress within the vascular wall—a notion supported by studies demonstrating its protective roles against hypertension and endothelial dysfunction [47]. This is particularly relevant considering that Ang II-induced signaling has been shown to enhance SOCE activity, partially through oxidative pathways, which exacerbates Ca^2+^ dysregulation in VSMC [48]. Collectively, these insights point toward a scenario where TRPM8 activation may provide a dual benefit: alleviating maladaptive Ca^2+^ signaling cascades while potentially mitigating oxidative injury in AAA.

It is also important to note that TRPC1 rarely functions in isolation but forms heteromeric complexes with other TRP channels, such as TRPC4 or TRPC5, which fundamentally influence channel properties and calcium entry specificity [49]. Therefore, it is plausible that TRPM8 activation modulates these channel assemblies, thereby fine-tuning calcium entry and vascular responsiveness [6].

### 4.4. Limitations and Future Perspectives

From a therapeutic standpoint, pharmacological modulators of SOCE channels have shown promise in attenuating vascular remodeling. Notably, inhibition of SOCE has been directly proven to attenuate the progression of AAA in animal models [50]. The present observations suggest that targeting TRPM8 could represent a novel strategy to regulate pathological SOCE and improve vascular function in AAA.

Several limitations of this study should be acknowledged. First, due to the use of cross-sectional sections for pathological analysis rather than longitudinal sections, we were unable to capture both the aneurysm and adjacent non-aneurysm regions within a single section from the same patient sample. Second, though we observed a functional interaction between TRPM8 and TRPC1, the precise molecular nature of their association remains unclear. We speculate this may involve competition for signaling lipids or a direct protein–protein interaction within a signaling complex, possibilities that warrant further biochemical investigation. Additionally, while the papain-induced model replicates key structural features of AAA, incorporating Ang II-infusion models in future studies would provide a more complete view, especially considering hypertensive contributions. Finally, validating these mechanistic insights in primary human vascular cells would strengthen the translational relevance of TRPM8 as a therapeutic target.

## 5. Conclusions

In conclusion, this study reveals dysregulation of TRP channels in AAA, highlighted by the upregulation of TRPC1, TRPC6, and TRPM8 in human and murine tissues. The paradoxical impairment of vasoconstriction despite elevated TRPC1, along with TRPC6-associated VSMC phenotypic switching, likely contributes to vascular dysfunction and aneurysm progression. Notably, TRPM8 activation potently inhibits TRPC1-mediated Ca^2+^ signaling, with amplified effects under pathological conditions, suggesting a possible protective mechanism. These observations provide preliminary evidence supporting further exploration of TRPM8 modulation as a potential approach to mitigate maladaptive calcium dysregulation and vascular remodeling in AAA, though additional studies are needed to confirm its therapeutic value.

## Data Availability

All additional data supporting the findings are provided in the Supplementary Information and are available from the corresponding author upon reasonable request.

## Supplementary Materials

The following supporting information can be downloaded at: https://www.mdpi.com/article/10.3390/biom16050741/s1, Figure S1: Concentration- and time-dependent effects of angiotensin II on human aortic smooth muscle cell (HASMC) proliferation; Figure S2: Validation of TRPC1 knockdown efficiency and functional assessment of store-operated calcium entry in HASMC. Table S1. Primer sequences. Supplementary Data S1. Source data for all figures. Supplementary Data S2. Partial raw data for isolated vessel ring contractility. Original images of Figure 1K, Figure 2N and Figure 5B,E can be found in supplementary materials.

## Abbreviations

The following abbreviations are used in this manuscript:

AAA	Abdominal Aortic Aneurysm
Ang II	Angiotensin II
ANOVA	Analysis of Variance
ARRIVE	Animal Research: Reporting of In Vivo Experiments
BCA	Bicinchoninic Acid
BSA	Bovine Serum Albumin
Ca^2+^	Calcium Ion
cDNA	Complementary DNA
CON	Control
CPA	Cyclopiazonic Acid
DAB	3,3′-Diaminobenzidine
DMEM	Dulbecco’s Modified Eagle Medium
DMSO	Dimethyl Sulfoxide
ECL	Enhanced Chemiluminescence
ECM	Extracellular Matrix
EVG	Elastica van Gieson
FBS	Fetal Bovine Serum
GAPDH	Glyceraldehyde-3-Phosphate Dehydrogenase
HE	Hematoxylin and Eosin
HASMC	Human Aortic Smooth Muscle Cell
HRP	Horseradish Peroxidase
IACUC	Institutional Animal Care and Use Committee
IHC	Immunohistochemistry
IHASMC	Immortalised Human Aortic Smooth Muscle Cell
[Ca^2+^]_i_	Intracellular Calcium Concentration
I_SOCC_	Store-Operated Calcium Currents
I–V	Current–Voltage
KCl	Potassium Chloride
La^3+^	Lanthanum Ion
MMPs	Matrix Metallopeptidases
mRNA	Messenger RNA
PBS	Phosphate-Buffered Saline
PE	Phenylephrine
PIP2	Phosphatidylinositol 4,5-Bisphosphate
PTEN	Phosphatase and Tensin Homolog
PVDF	Polyvinylidene Difluoride
RhoA	Ras Homolog Gene Family Member A
RIPA	Radioimmunoprecipitation Assay
ROCK	Rho-Associated Coiled-Coil Containing Protein Kinase
ROCE	Receptor-Operated Calcium Entry
RT-qPCR	Real-Time Quantitative Polymerase Chain Reaction
SD	Standard Deviation
SDS-PAGE	Sodium Dodecyl Sulfate–Polyacrylamide Gel Electrophoresis
siNC	Small Interfering RNA Negative Control
siRNA	Small Interfering RNA
siTRPC1	Small Interfering RNA Targeting TRPC1
SOCE	Store-Operated Calcium Entry
SOCC	Store-Operated Calcium Channel
TBST	Tris-Buffered Saline with Tween 20
TGFβ	Transforming Growth Factor Beta
TRP	Transient Receptor Potential
TRPC1	Transient Receptor Potential Canonical 1
TRPC6	Transient Receptor Potential Canonical 6
TRPM8	Transient Receptor Potential Melastatin 8
TRPV4	Transient Receptor Potential Vanilloid 4
VSMC	Vascular Smooth Muscle Cell
