# Supplementary material for: Up-Regulation of the TRPM8 Channel Attenuates TRPC1-Mediated Store-Operated Calcium Entry in Abdominal Aortic Aneurysm
Source: Biomolecules. 2026 May 19;16(5):741. doi: 10.3390/biom16050741 (PMC13204588; doi:10.3390/biom16050741)
Supplement: Supplementary file 1 [file biomolecules-16-00741-s001.zip › Supplementary Material-figures.pdf]

Supplementary Material

Up-regulation of the TRPM8 Channel Attenuates TRPC1-Mediated Store-Operated Calcium Entry in Abdominal Aortic Aneurysm

Yi-Qian Wang[1,3a], Min Pan[1,3a], Yi-Chen Lin,[3,5,6], Si-Yi Zheng[3], Qing-Ye Chen[1,3], Long-Xin Gui[1,3], Mo-Jun Lin[1,3]* and Da-Cen Lin[2,3,4]*

[1]*Department of Physiology and Pathophysiology, School of Basic Medical Sciences, Fujian Medical University, Fuzhou, 350122, PR China*

[2]*Department of Epidemiology and Health Statistics,School of Public Health, Fujian， Medical University, Fuzhou, 350122, PR China*

[3]*Key Laboratory of Fujian Province Universities on Ion Channel and Signal Transduction in Cardiovascular Diseases, Fujian Medical University, Fuzhou, 350122, PR China*

[4]*NHC Key Laboratory of Etiological Epidemiology of Chronic Diseases with High Incidence in Fujian-Taiwan Area (Co-construction), Fujian Medical University, Fuzhou 350122, PR China*

[5]*Department of Vascular Surgery, The First Affiliated Hospital, Fujian Medical University, Fuzhou, 350005, PR China*

[6]*Department of Vascular Surgery, National Regional Medical Center, Binhai Campus of the First Affiliated Hospital, Fujian Medical University, Fuzhou, 350212, PR China*

*Correspondence: D.-C. Lin, ldc@fjmu.edu.cn; or M.-J. Lin, mjlin@fjmu.edu.cn.

[a]Y.-Q. Wang, M. Pan contributed equally.

Supplementary Figure

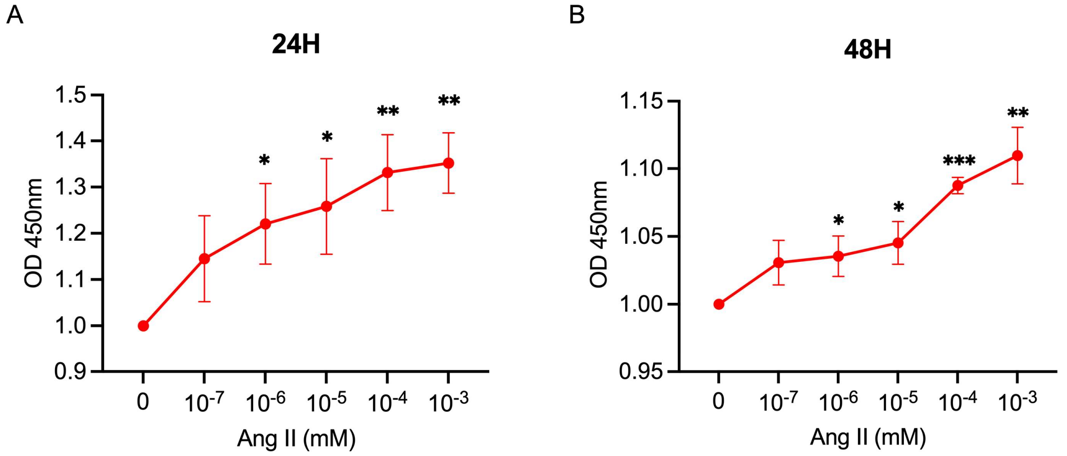

Figure S1. Concentration- and time-dependent effects of angiotensin II on human aortic smooth muscle cells (HASMCs) proliferation.

(A, B) CCK-8 proliferation assays of HASMCs treated with vehicle or increasing concentrations of angiotensin II (Ang II; 10^{-10}–10^{-6} mol/L) for 24 h (A) and 48 h (B). Data are presented as mean ± SD; n = 3. *P<0.05, **P<0.01, ***P<0.001 vs Vehicle.

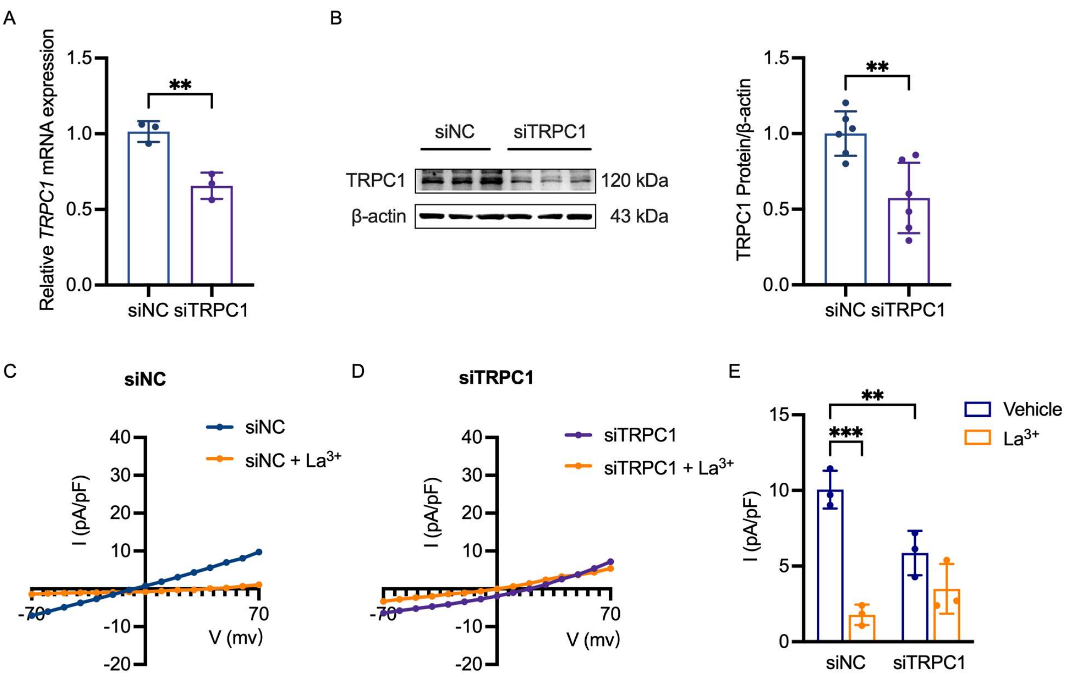

Figure S2. Validation of TRPC1 knockdown efficiency and functional assessment of store-operated calcium entry in HASMCs.

(A) RT–qPCR analysis showing the relative mRNA expression of TRPC1 in HASMCs transfected with TRPC1-specific siRNA (siTRPC1) compared to negative control siRNA (siNC). (B) Representative Western blot images (left) and corresponding densitometric quantification (right) of TRPC1 protein levels in siNC- and siTRPC1-transfected HASMCs. (C, D) Current–voltage (I–V) relationships obtained from whole-cell patch-clamp recordings illustrating the effects of La^{3+} on CPA-induced currents in siNC (C) and siTRPC1 (D) groups. (E) Summary of current densities (pA/pF) under the indicated experimental conditions. Data are presented as mean ± SD; n = 3 (A,E) and n = 6 (B). *P<0.05, **P<0.01, ***P<0.001.
