# Supplementary material for: Up-Regulation of the TRPM8 Channel Attenuates TRPC1-Mediated Store-Operated Calcium Entry in Abdominal Aortic Aneurysm
Source: Biomolecules. 2026 May 19;16(5):741. doi: 10.3390/biom16050741 (PMC13204588; doi:10.3390/biom16050741)

Human-TRPM8 in Fig.1 (230711)

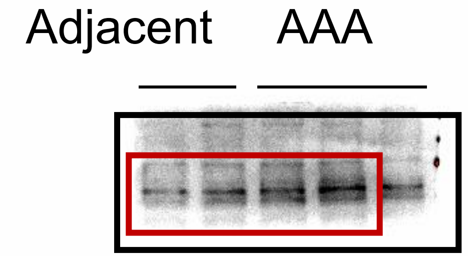

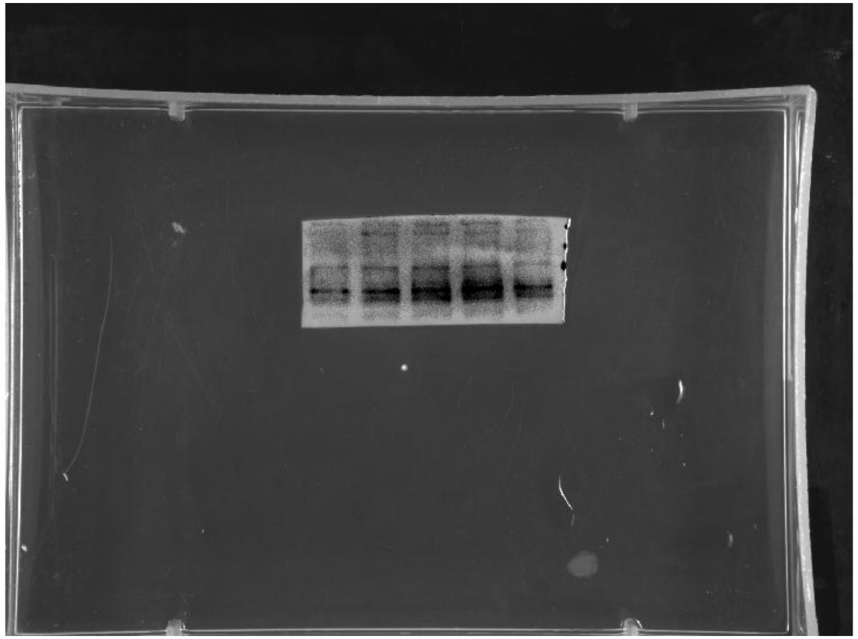

Human-actin

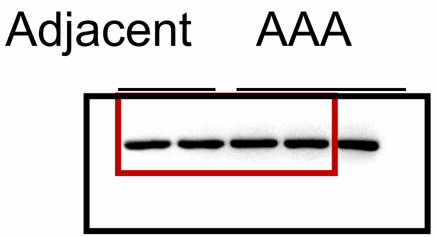

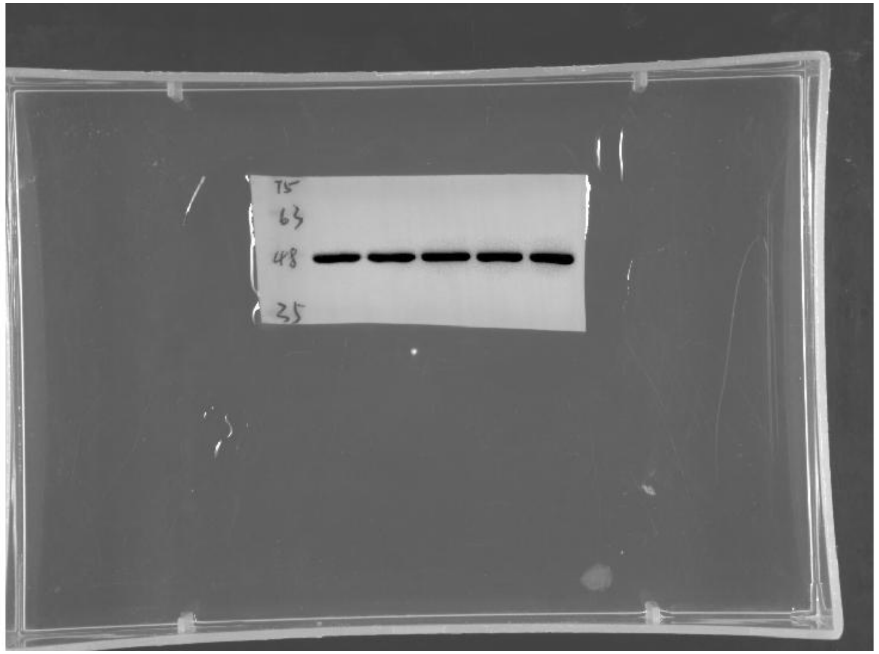

Human-TRPM8 (240113)

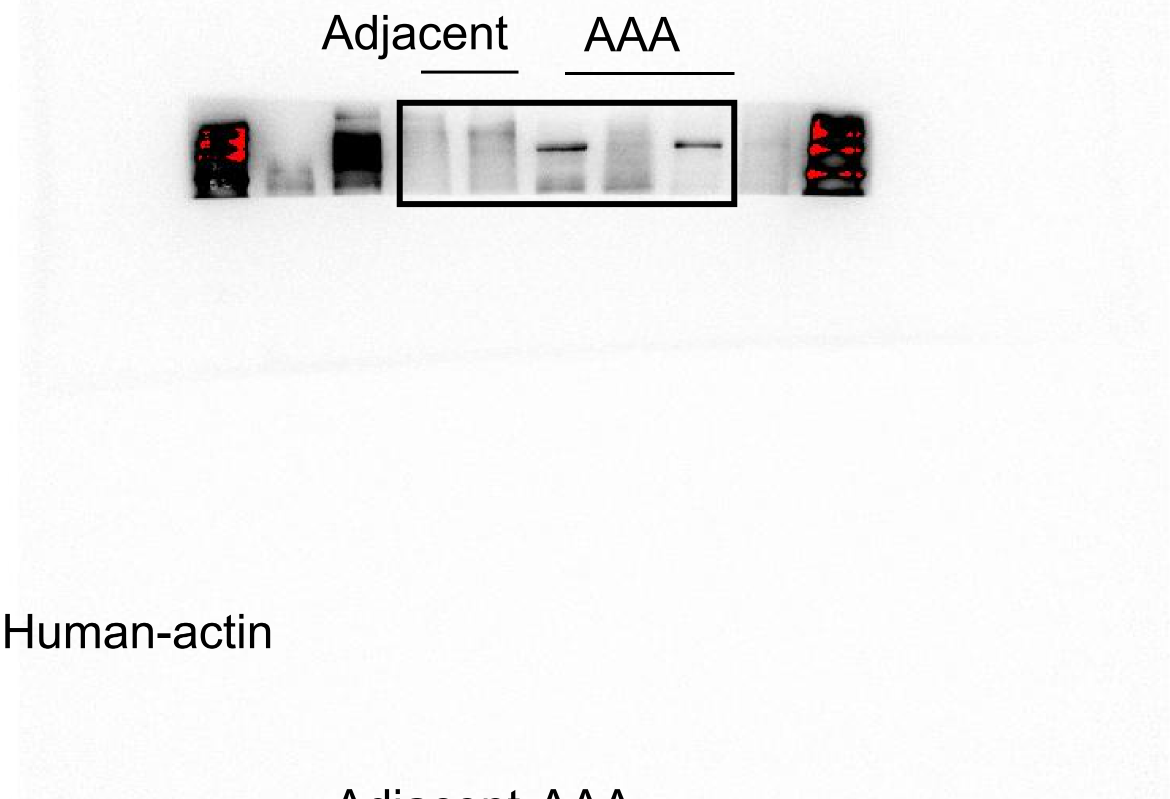

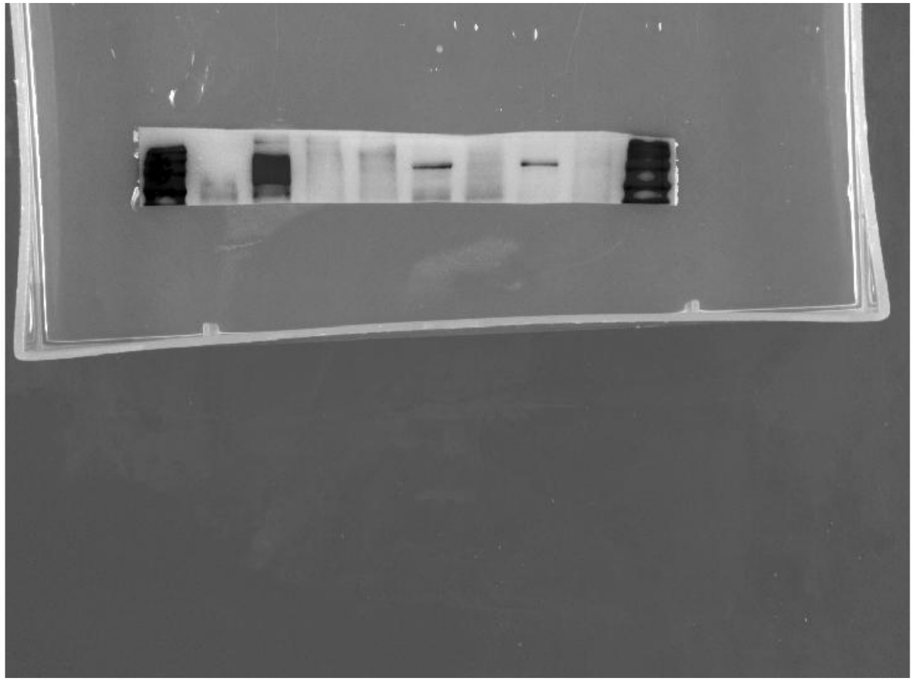

Human-actin

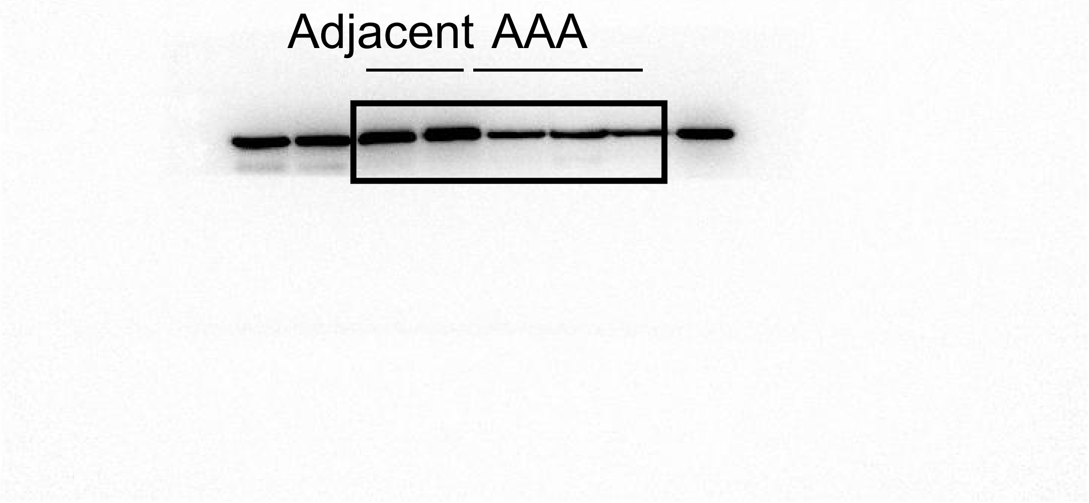

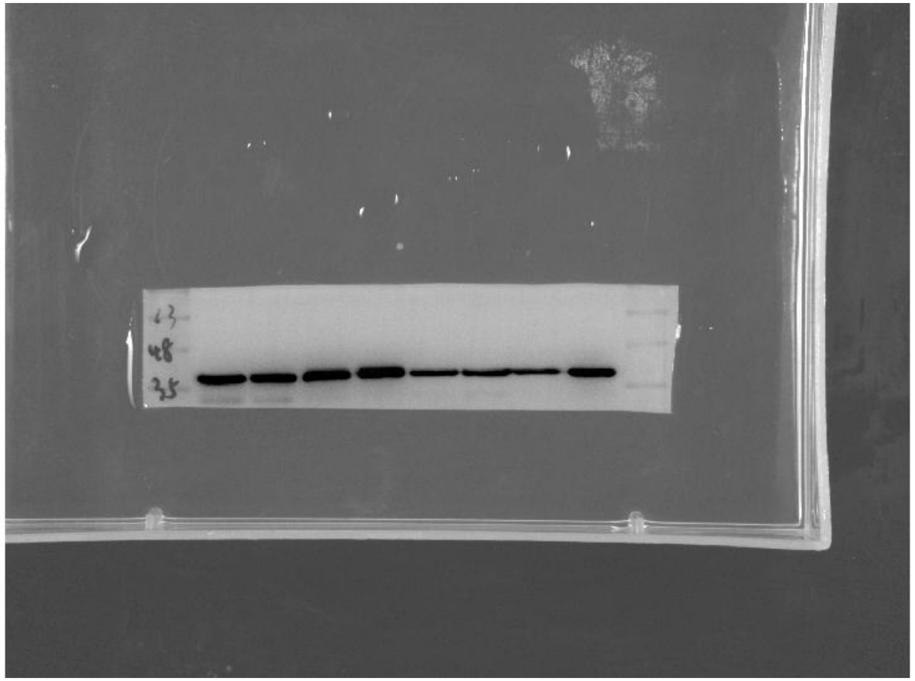

Human-TRPC1 in Fig.1 (230422)

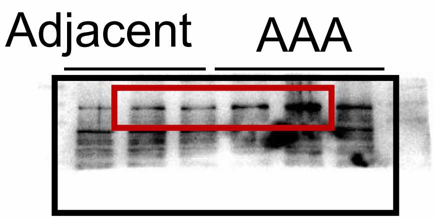

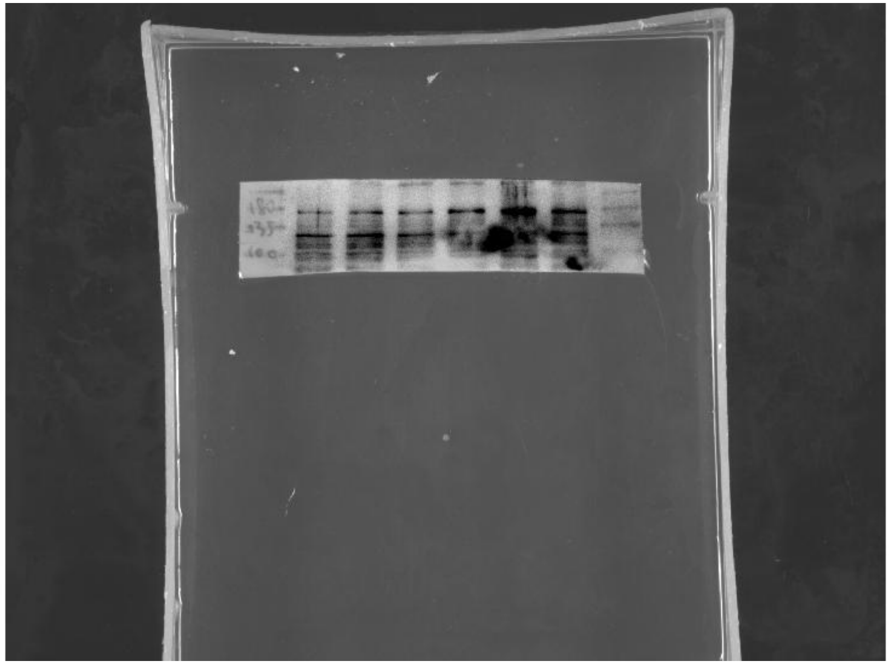

Human-actin

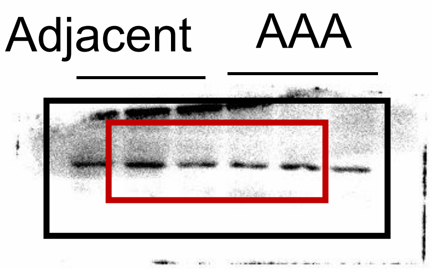

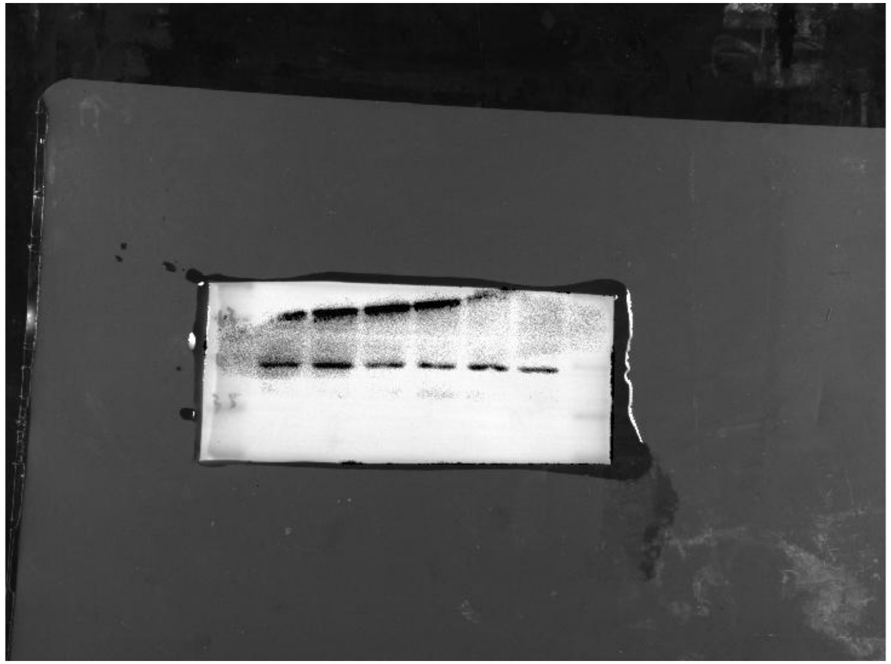

Human-TRPC1 (230711)

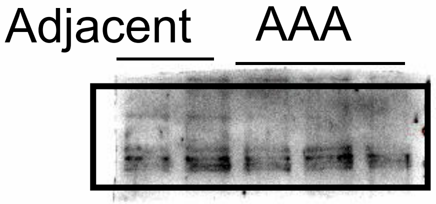

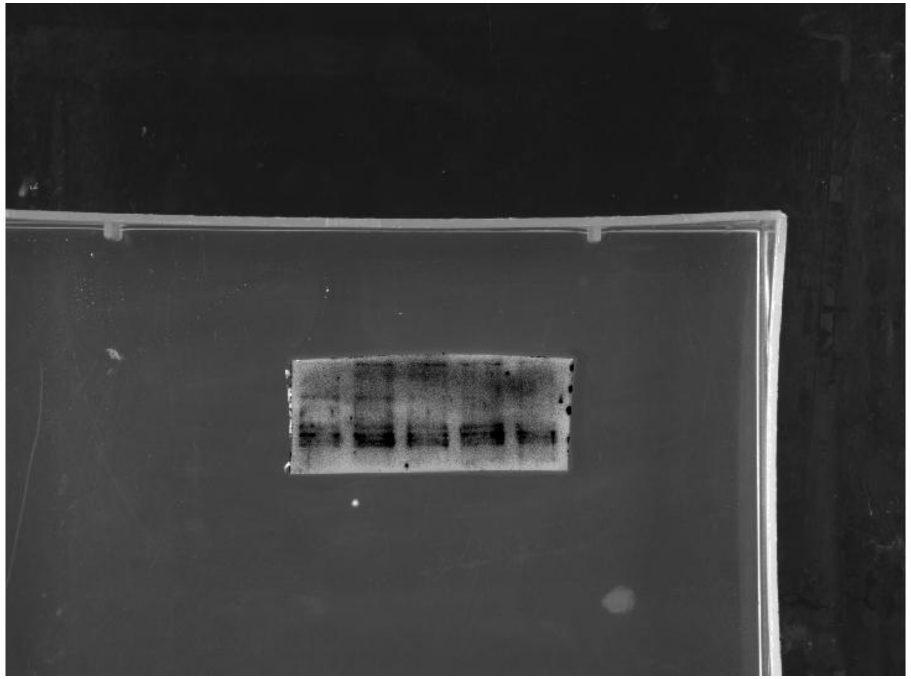

Human-actin

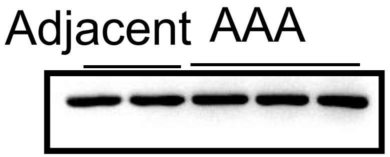

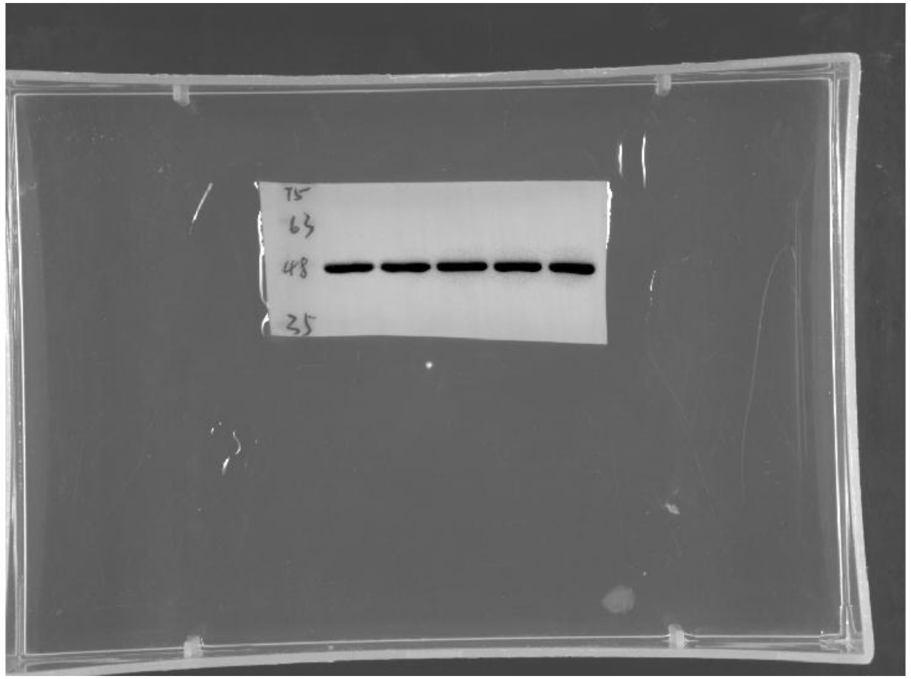

Mouse-TRPC1 in Fig.2 (231001)

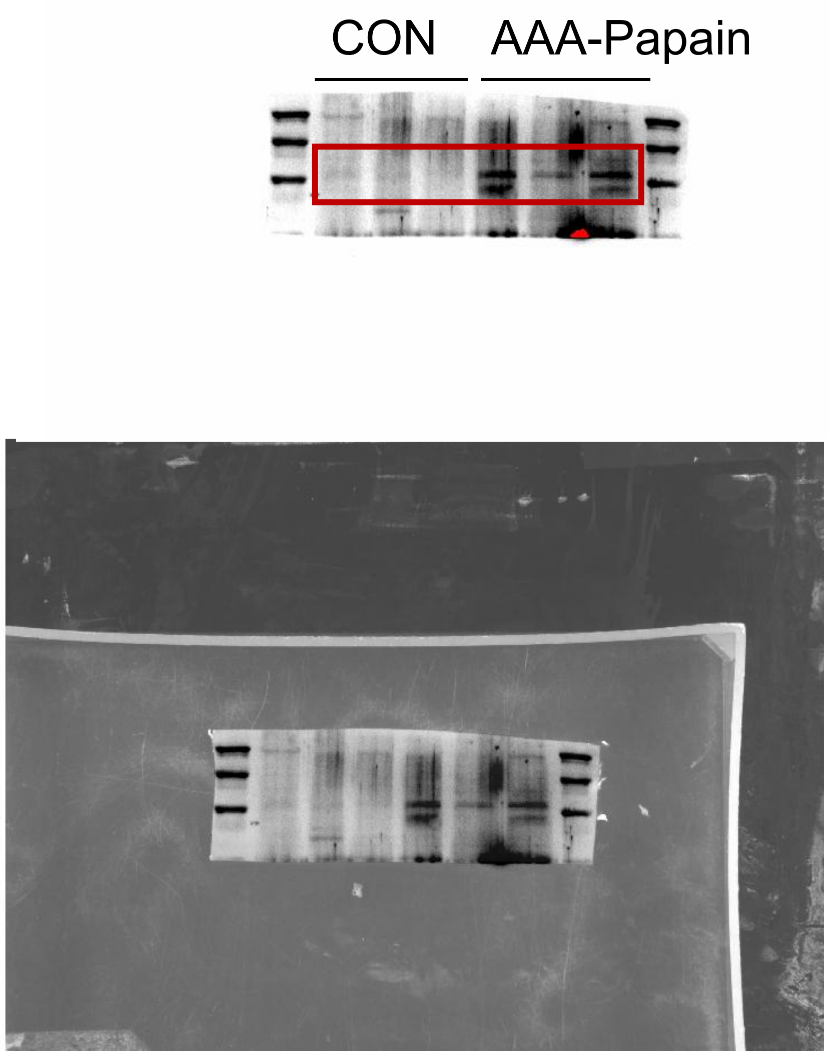

Mouse-TRPM8

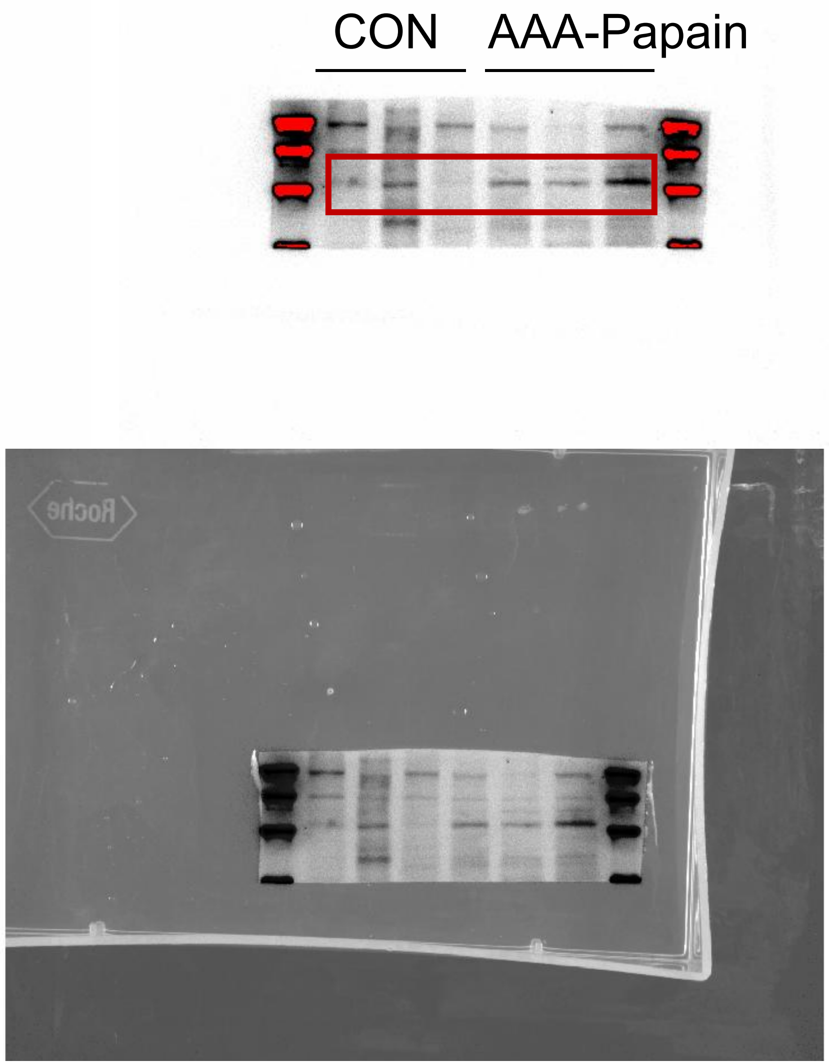

Mouse-actin

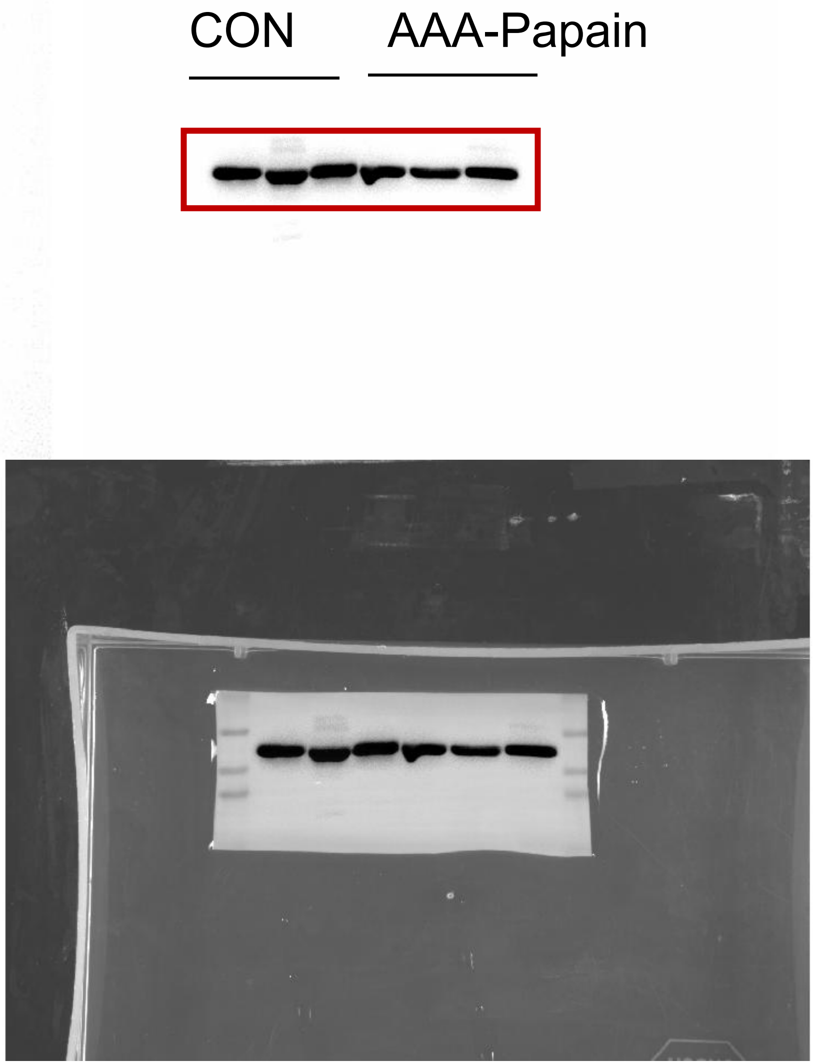

Mouse-TRPC1 (221111)

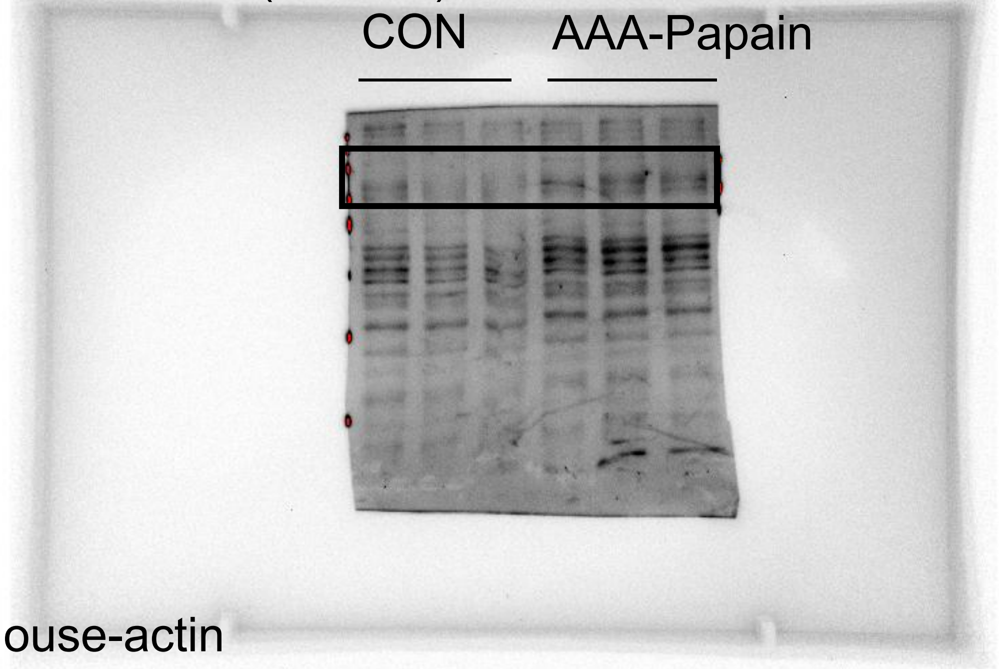

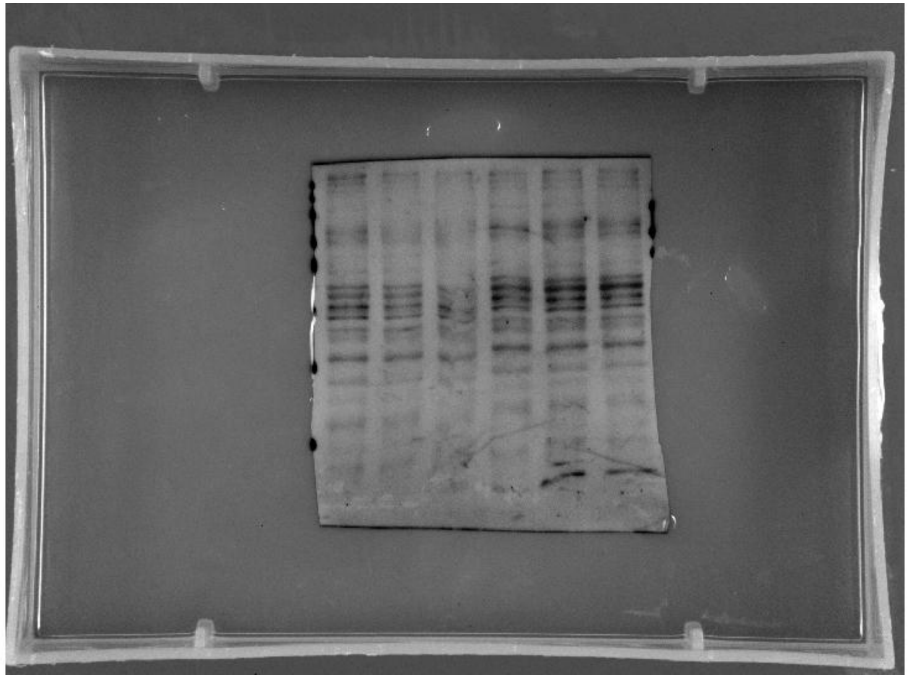

Mouse-actin

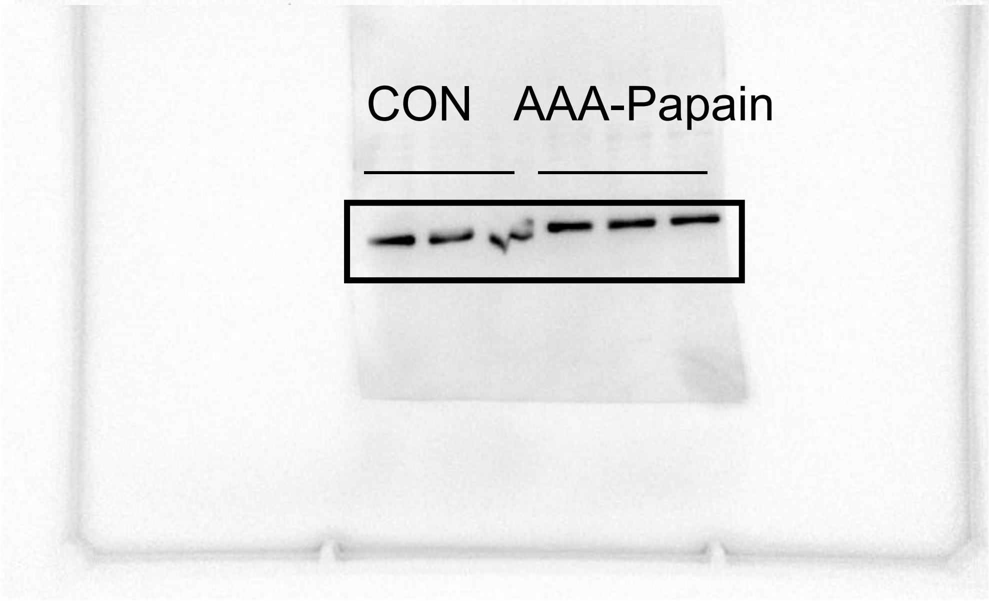

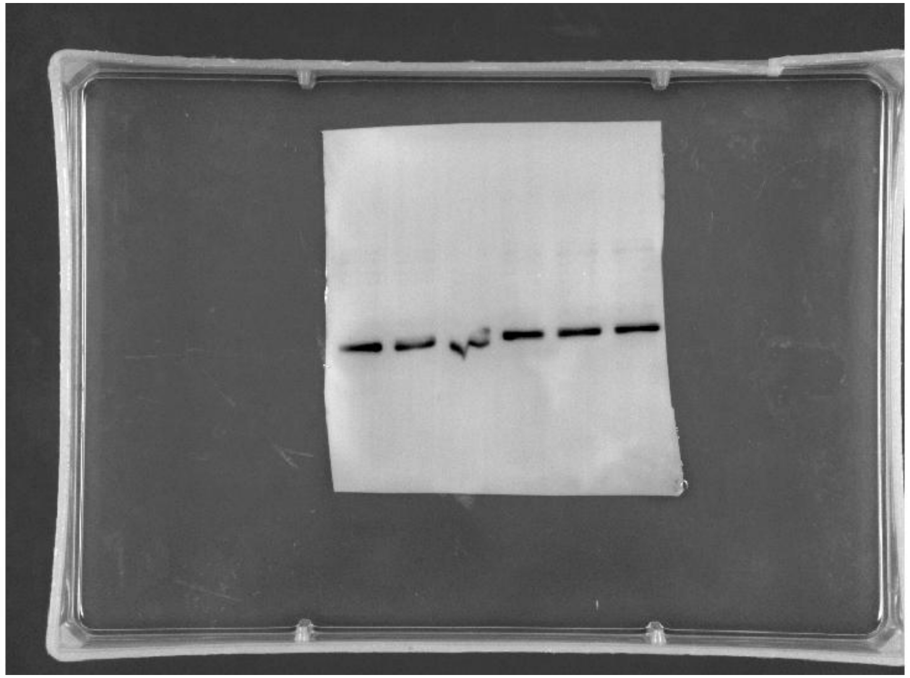

Mouse-TRPM8 (221115)

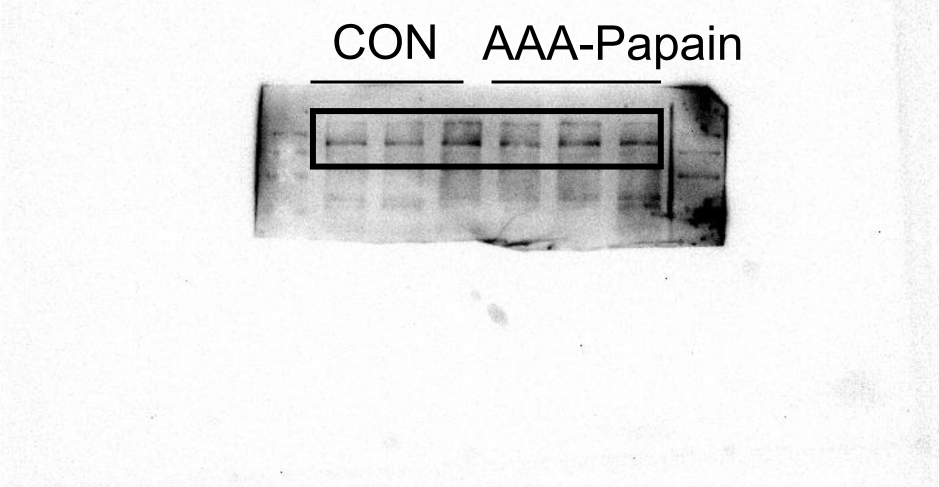

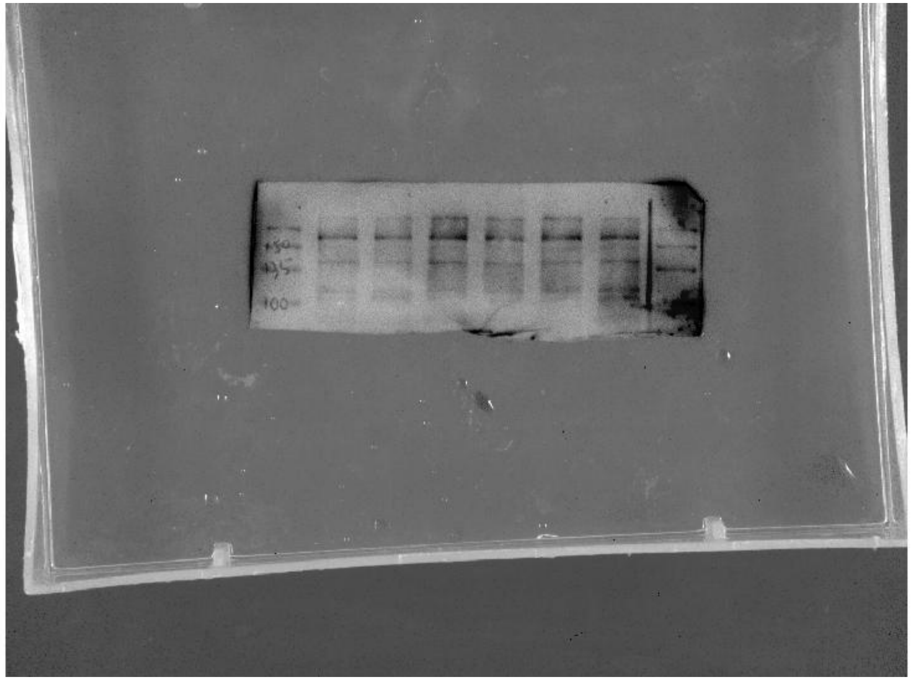

Mouse-actin

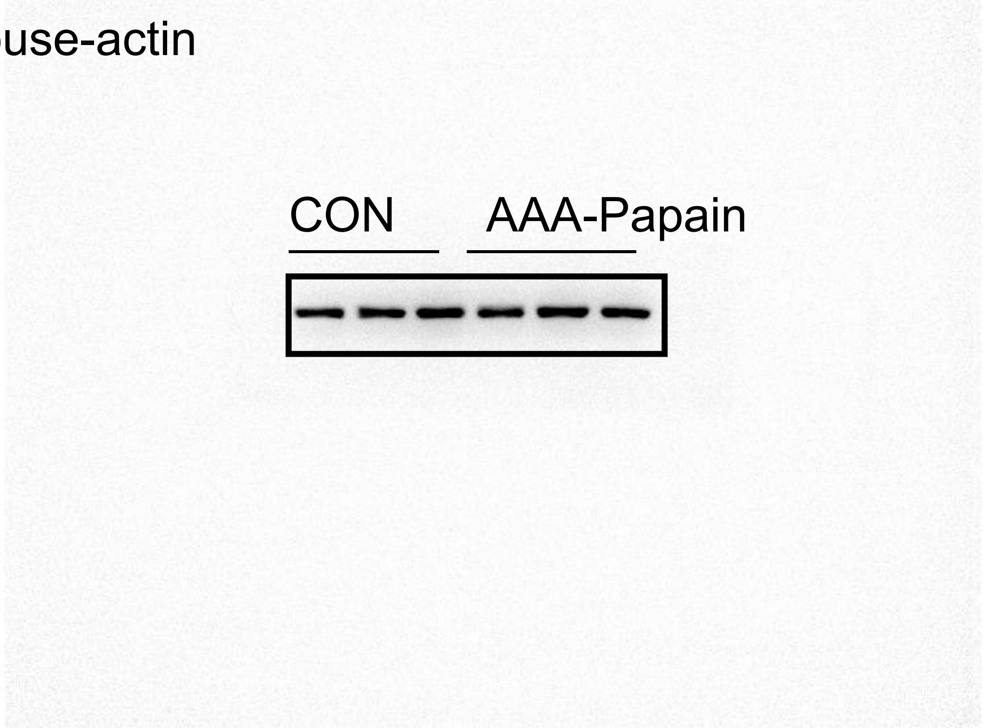

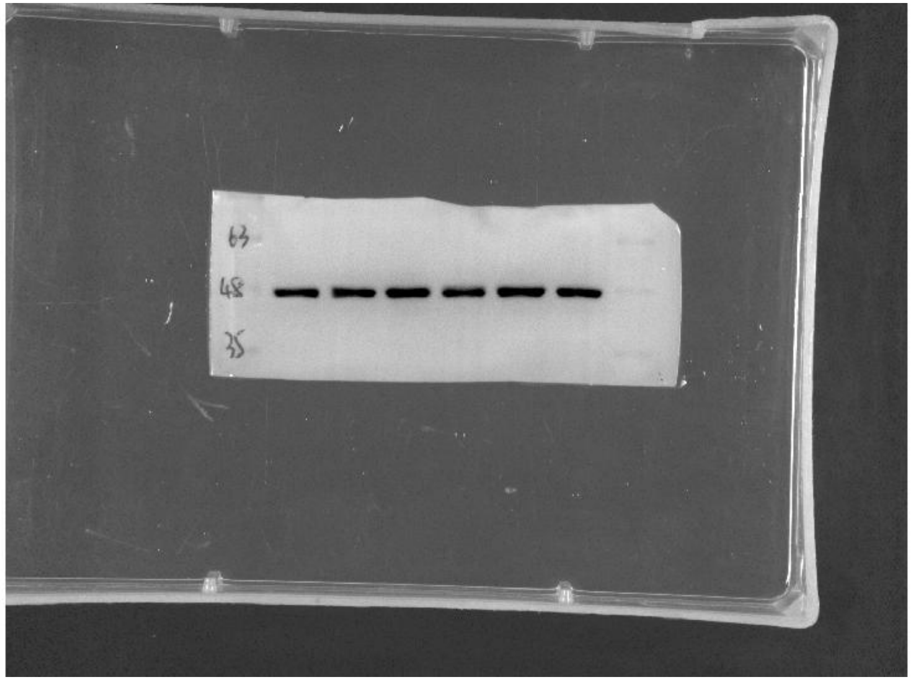

HASMC-TRPC1 in Fig.5B (230222-n2)

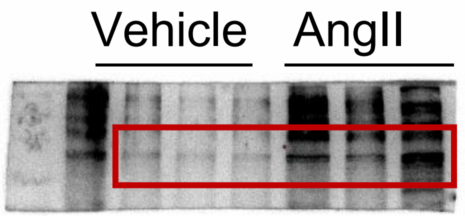

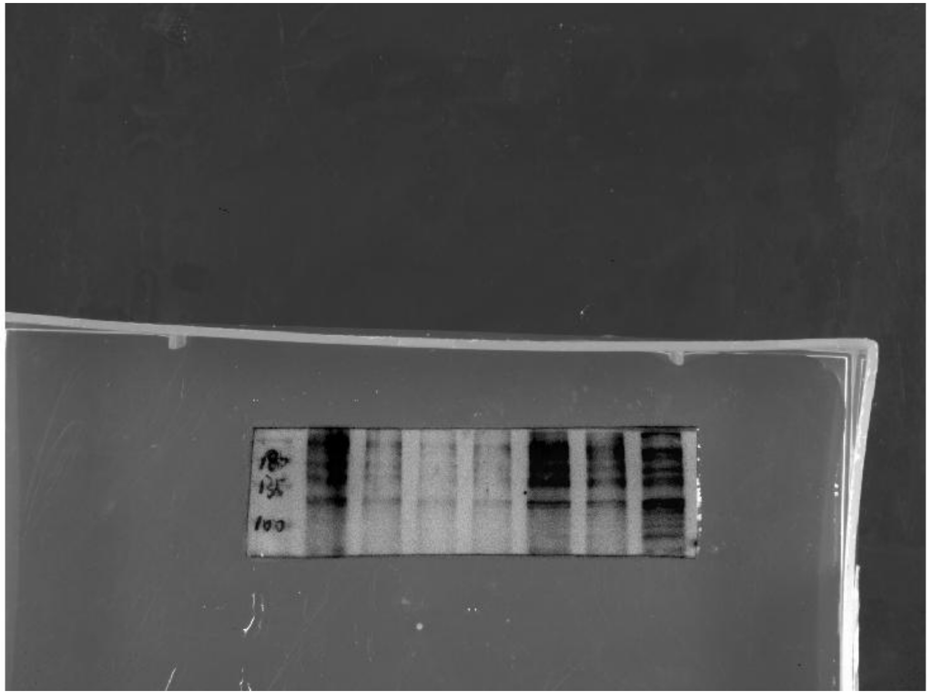

HASMC-actin

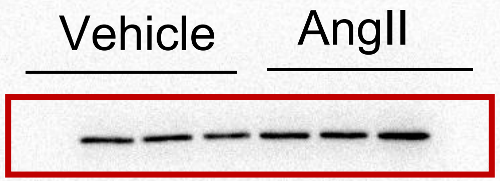

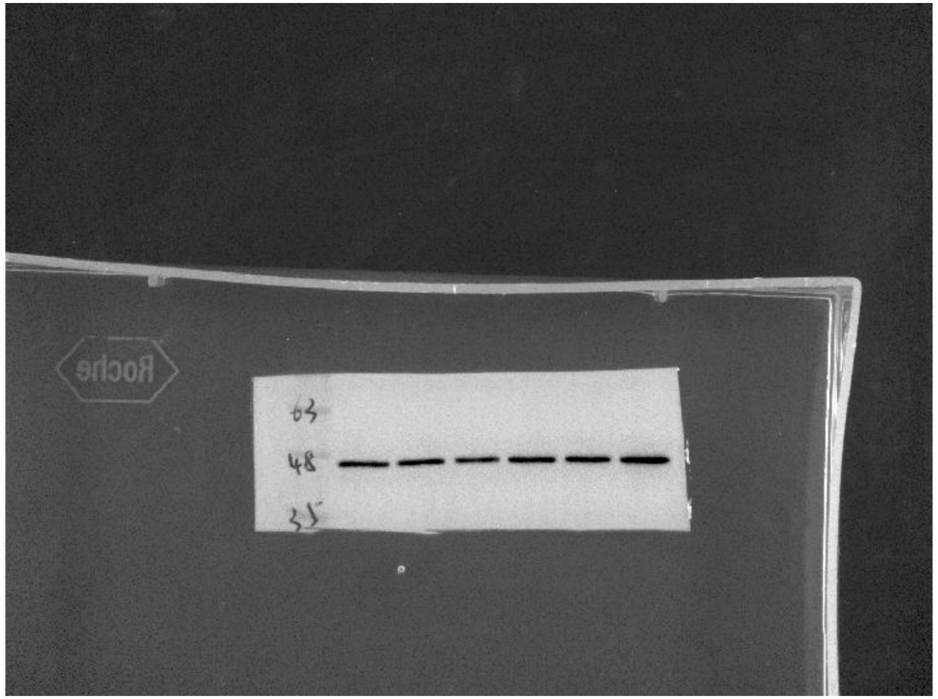

HASMC-TRPM8 in Fig.5B (221129-n8)

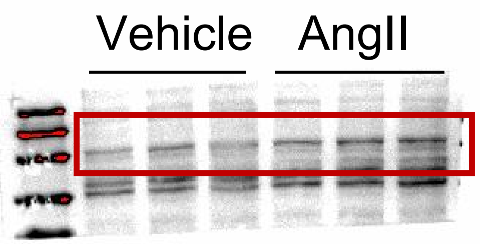

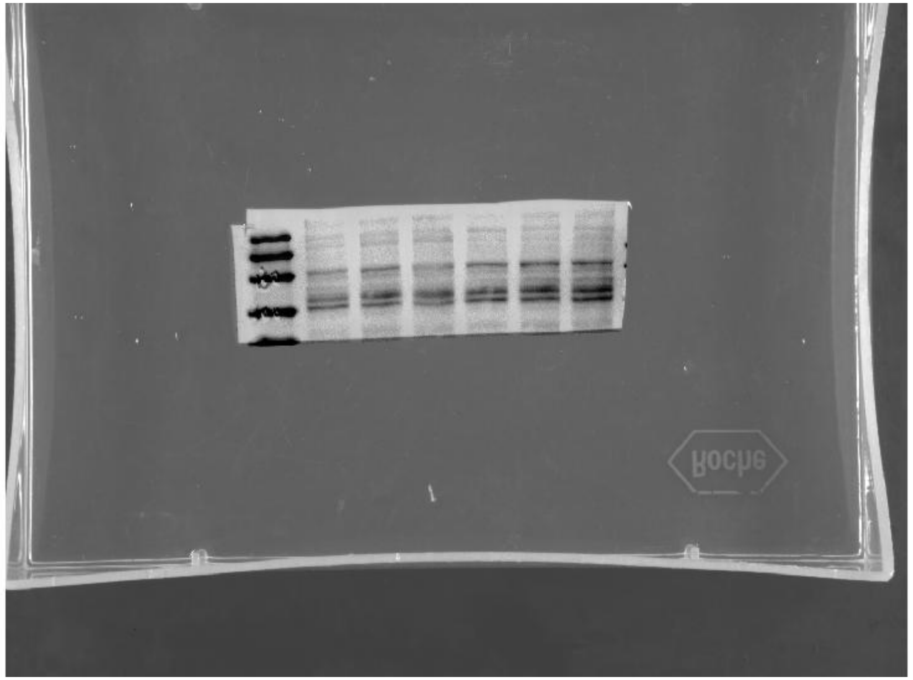

HASMC-actin

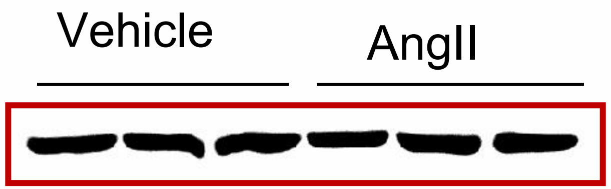

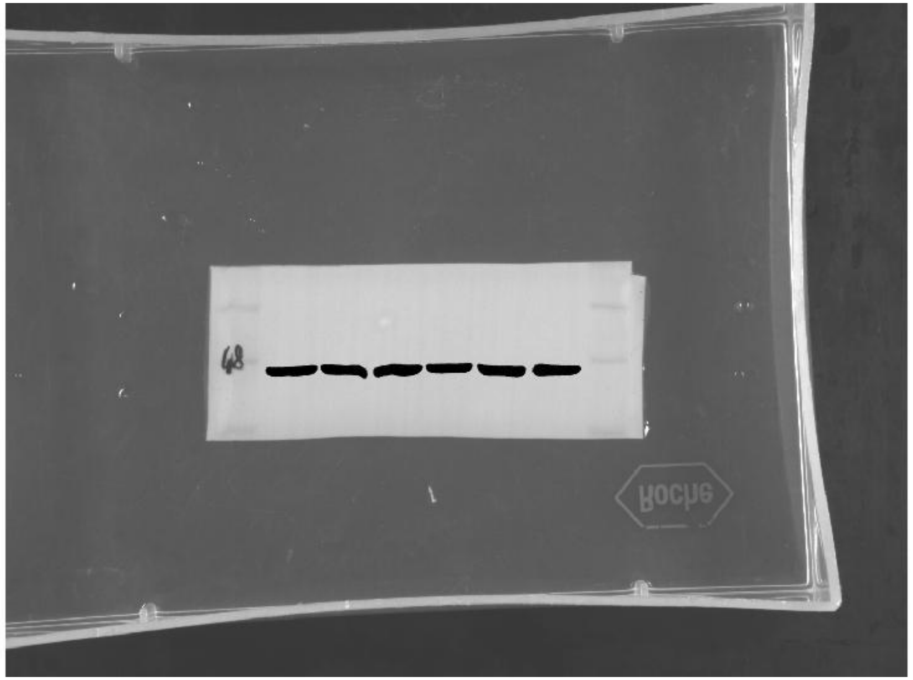

HASMC-TRPC1 (230218)

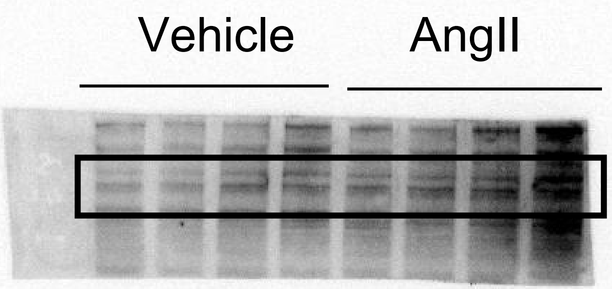

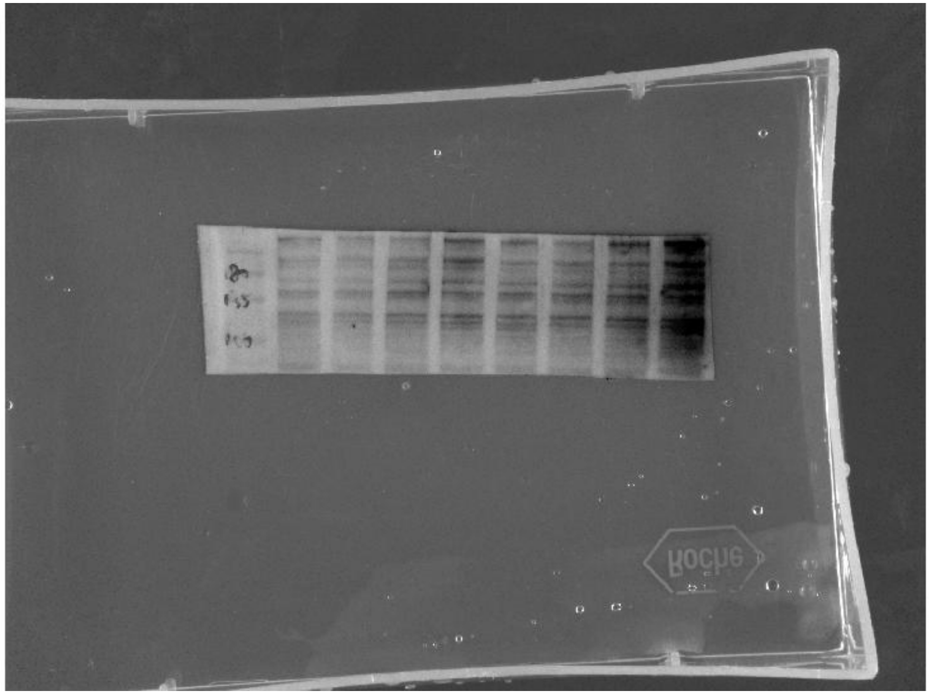

HASMC-actin

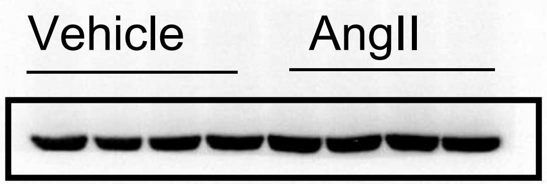

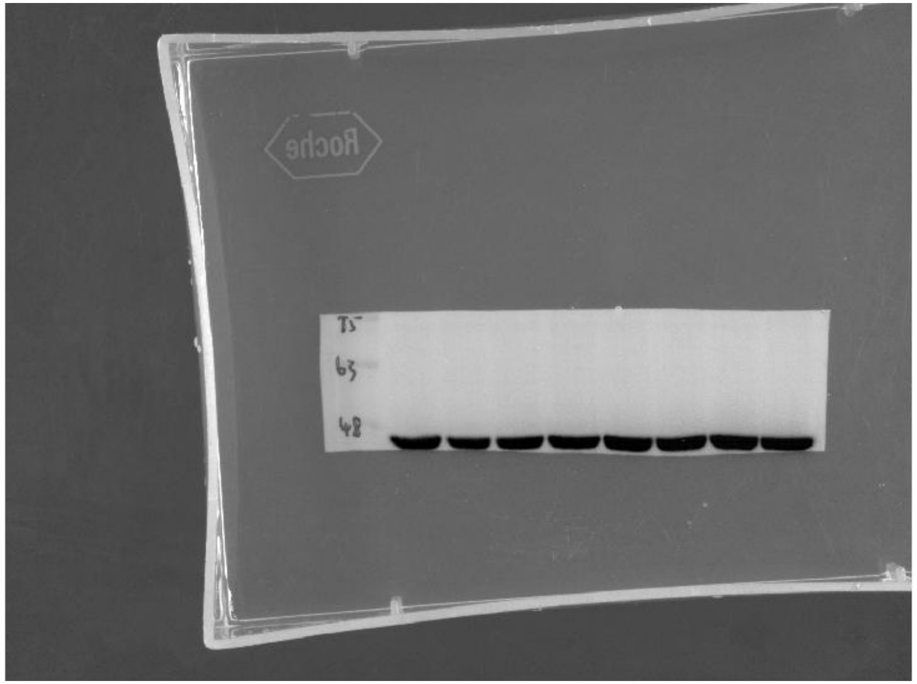

HASMC-TRPM8 (221129-n7)

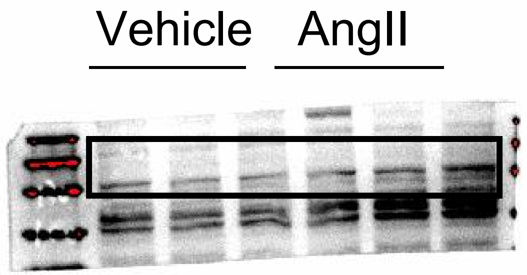

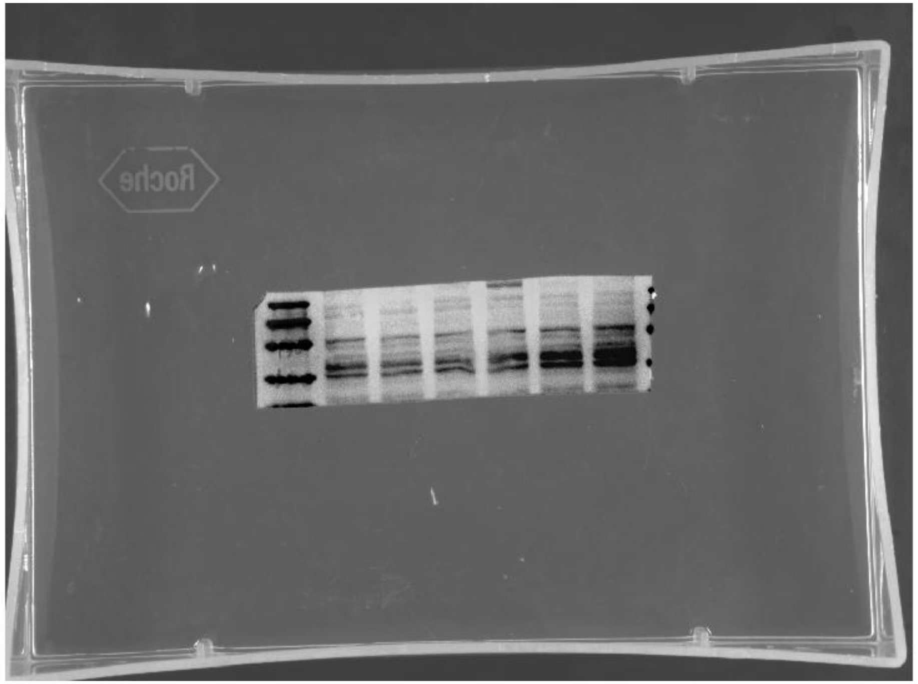

HASMC-actin

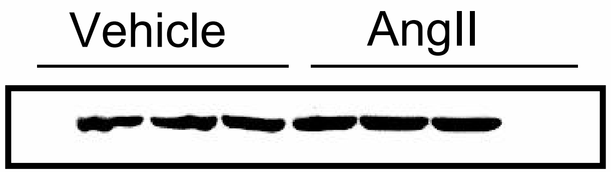

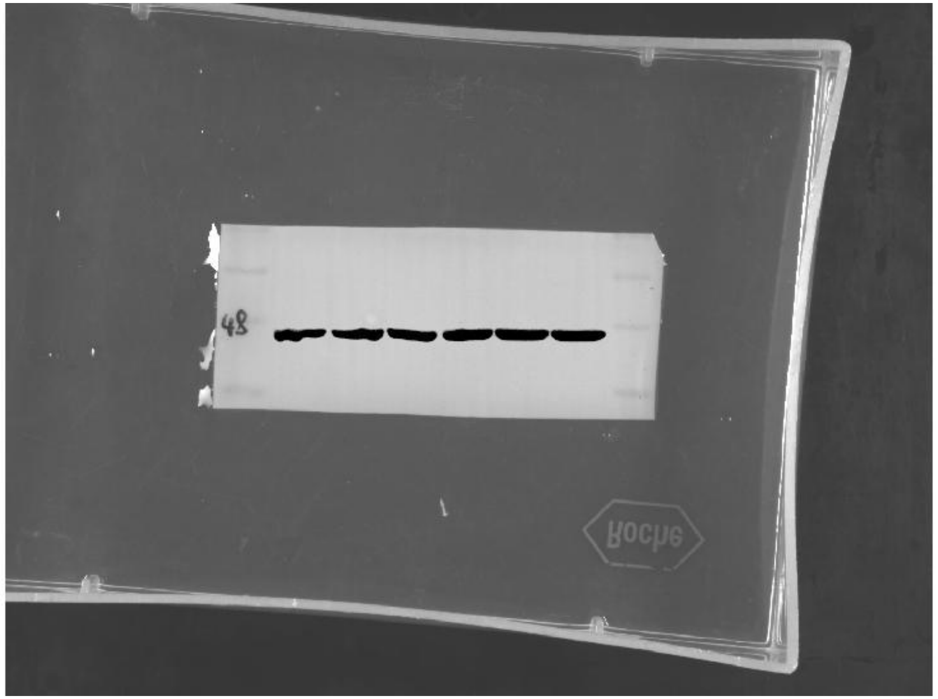

HASMC-TRPC1 in Fig.5E (260420-n2)

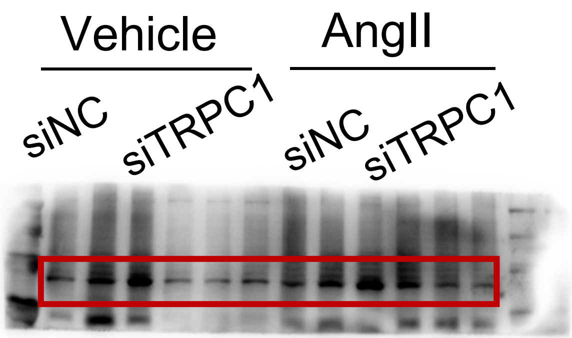

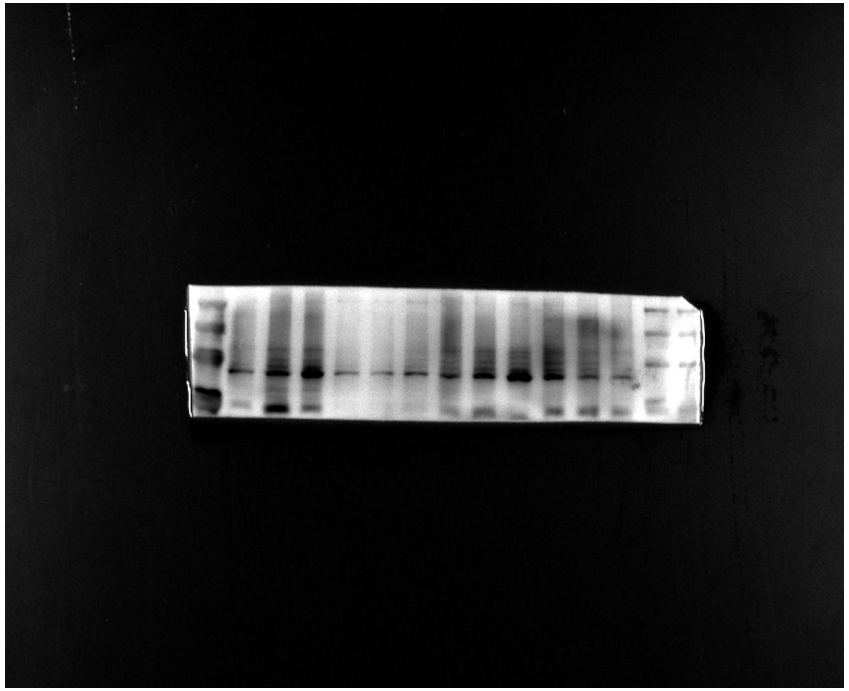

HASMC-actin

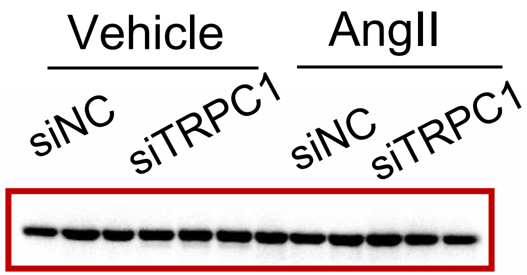

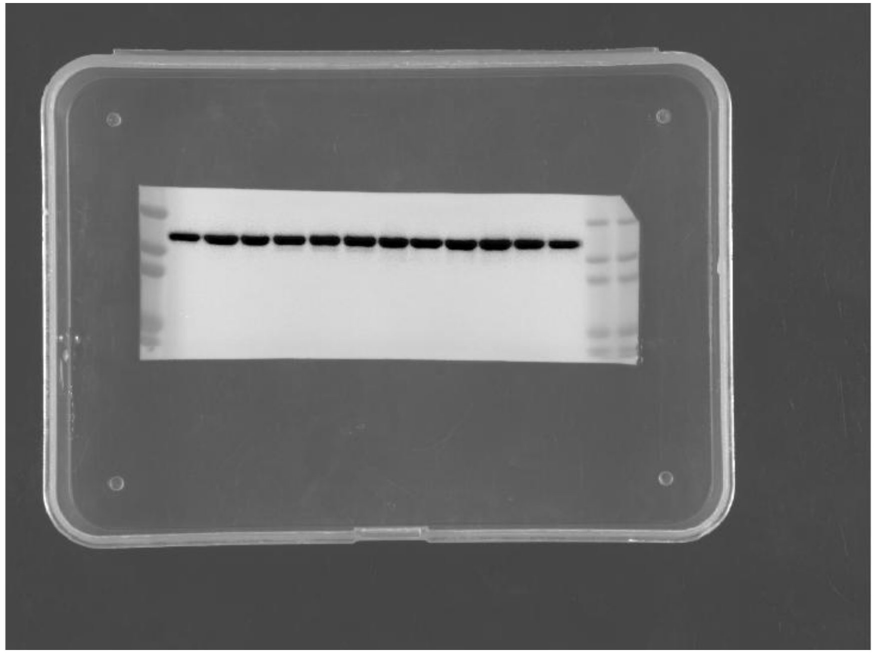

HASMC-TRPM8 in Fig.5E (260420-n1)

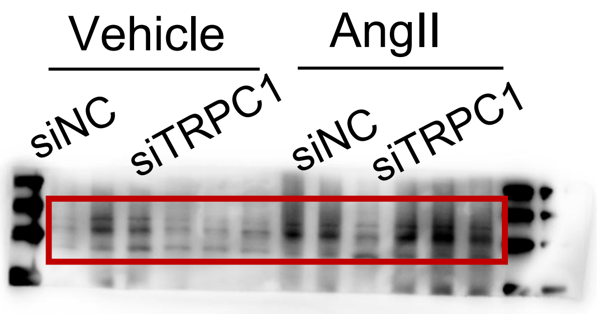

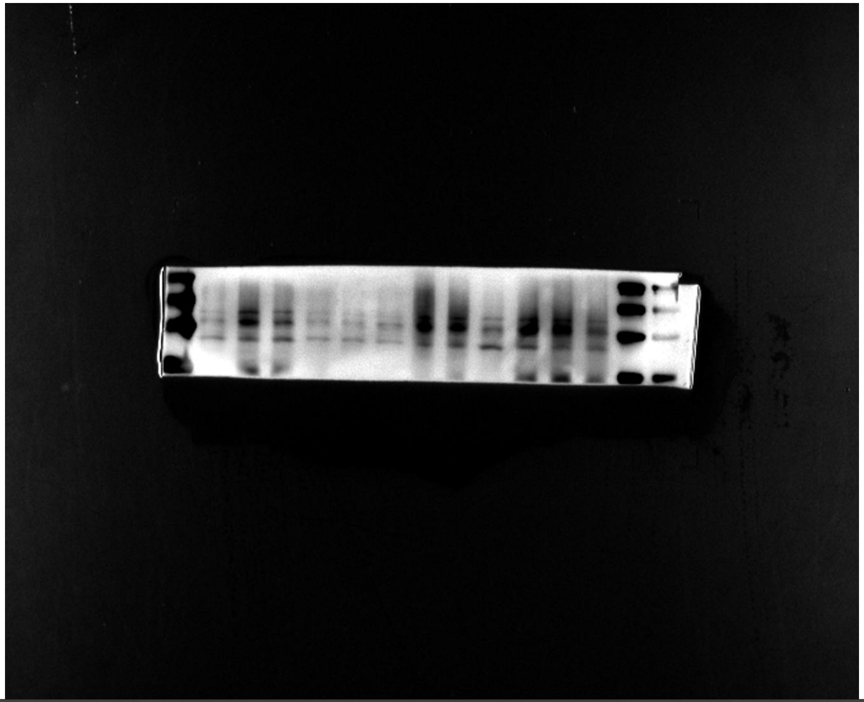

HASMC-actin

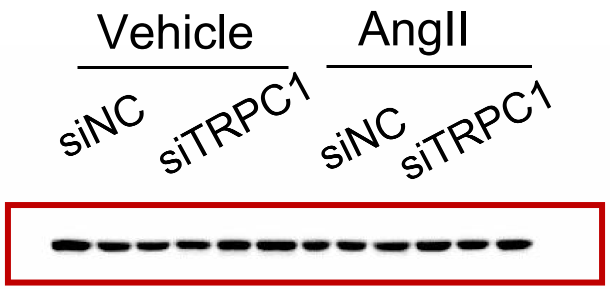

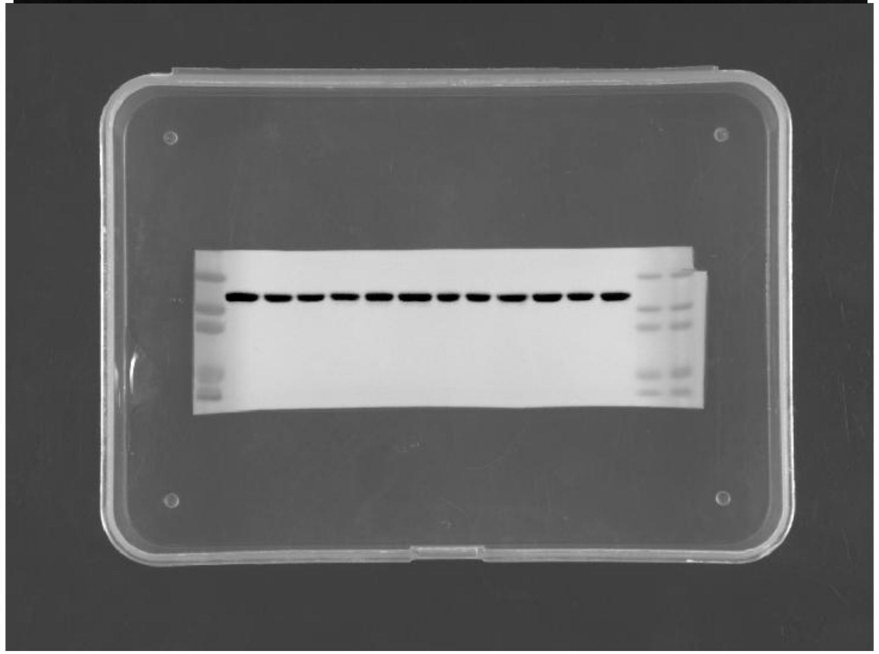

HASMC-TRPC1 (260420-n1)

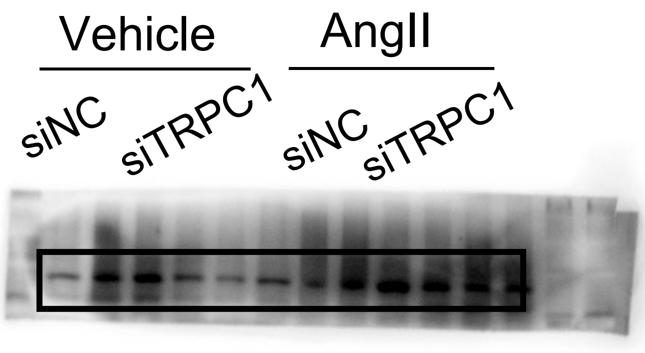

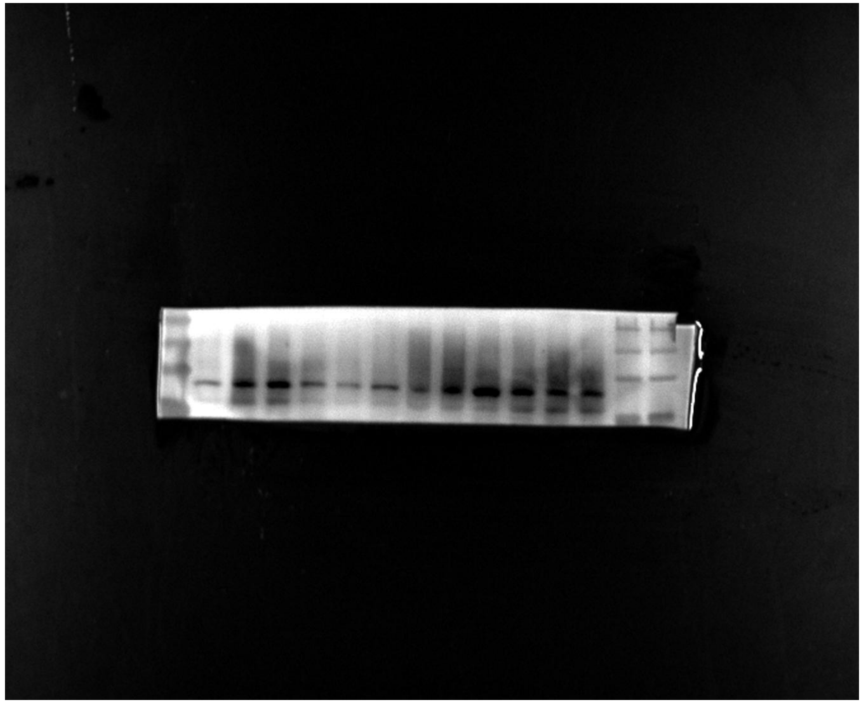

HASMC-actin

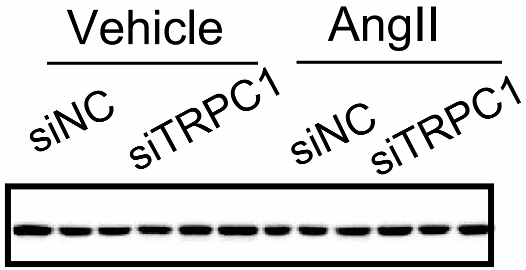

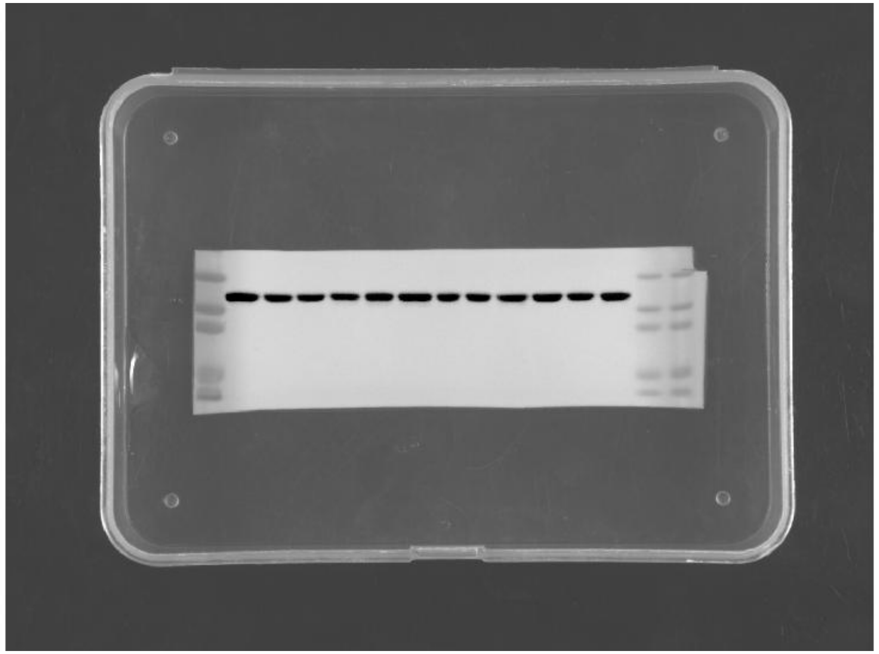

HASMC-TRPM8 (260420-n2)

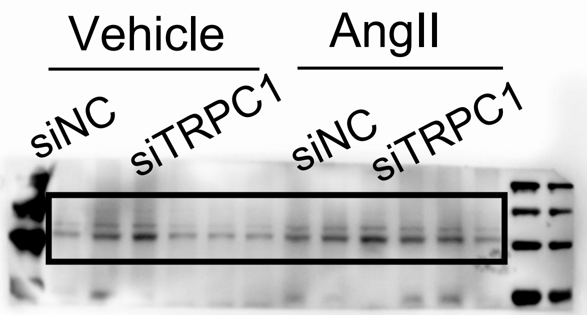

HASMC-actin

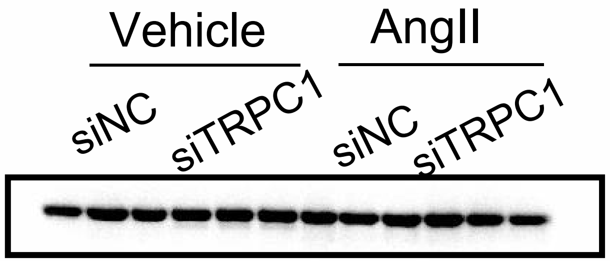

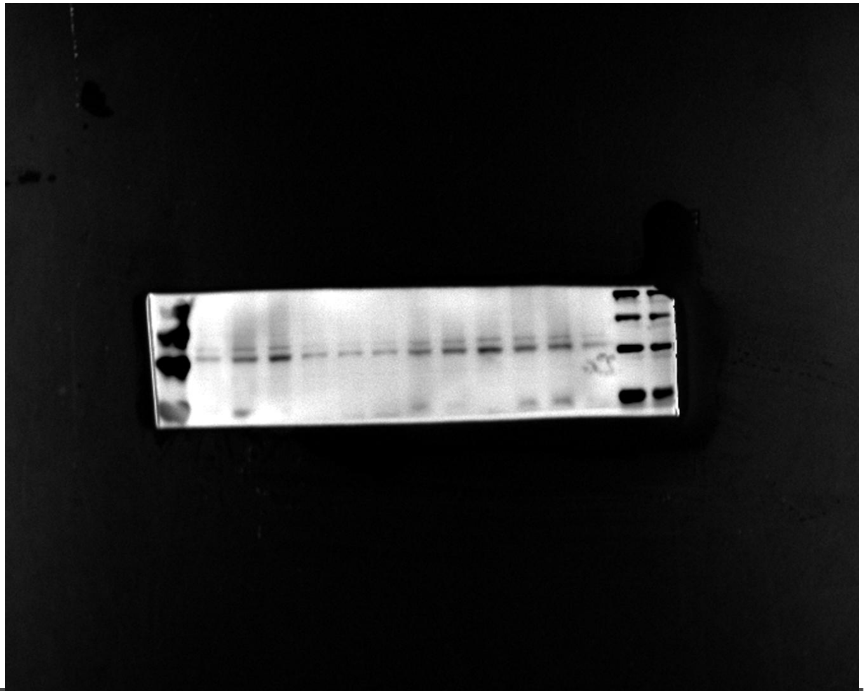

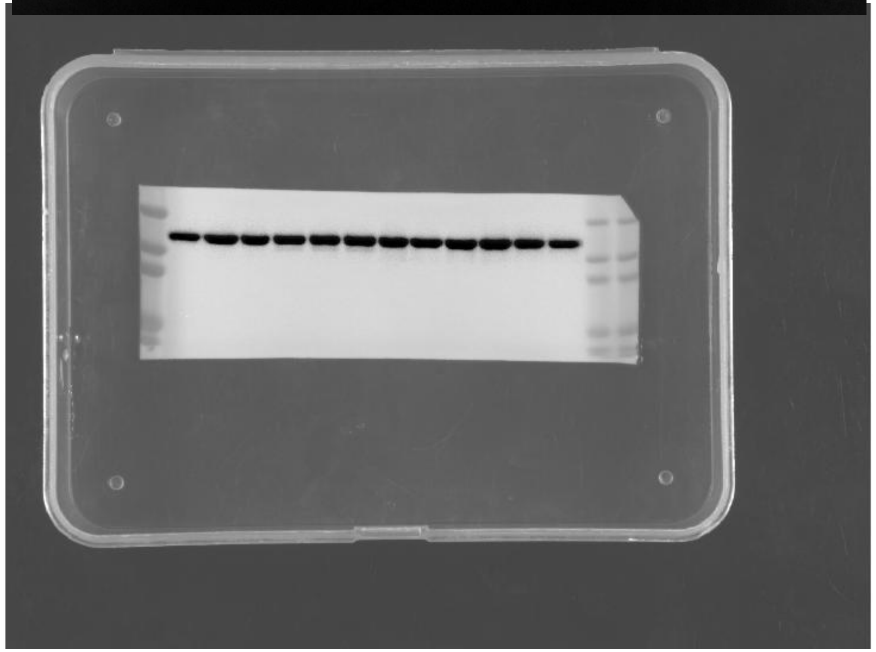

HASMC-TRPC1 in Fig.S2B (260331-n2)

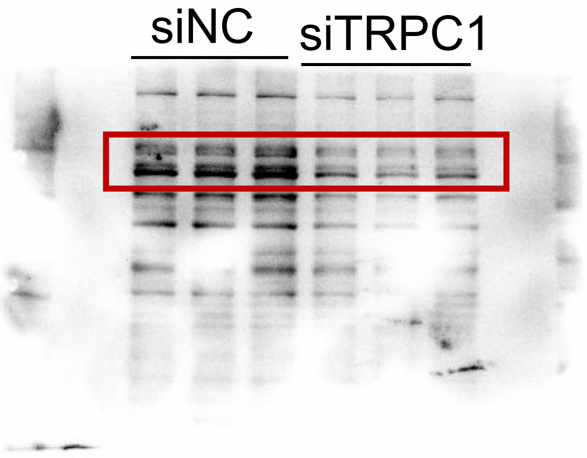

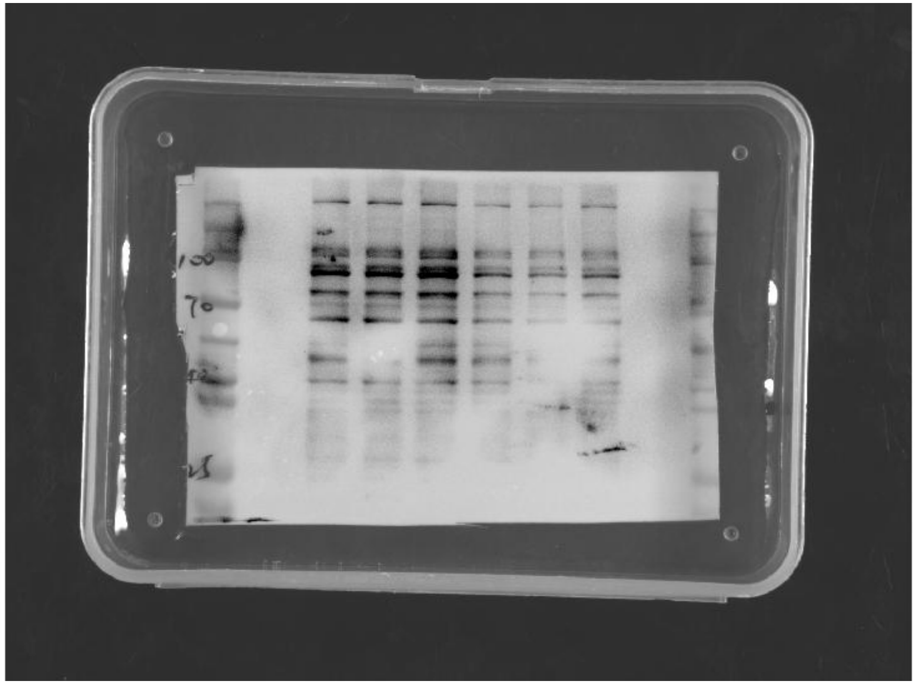

HASMC-actin

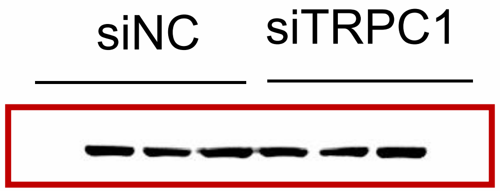

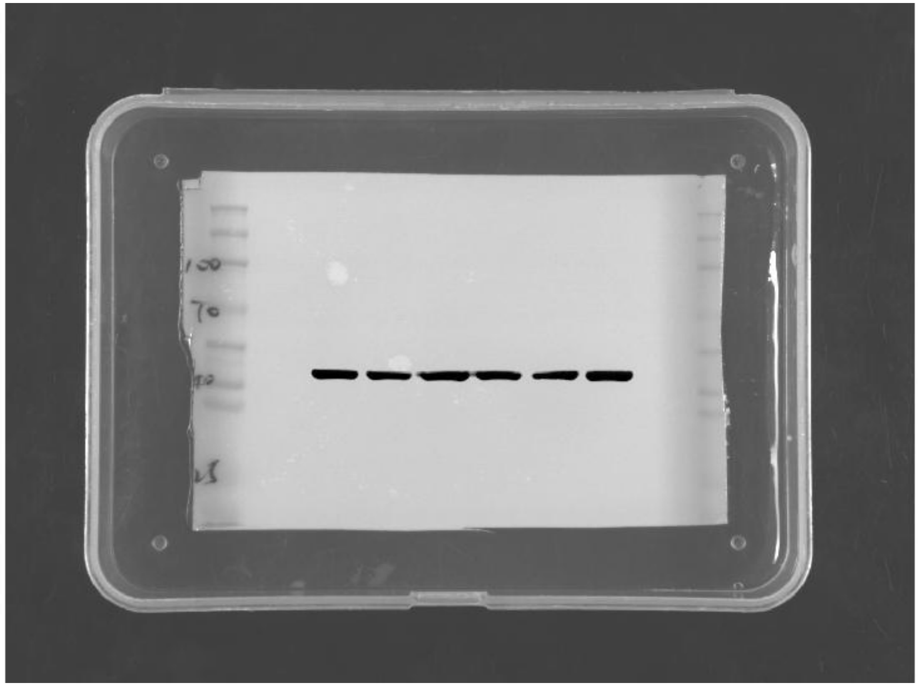

HASMC-TRPC1 (260331-n1)

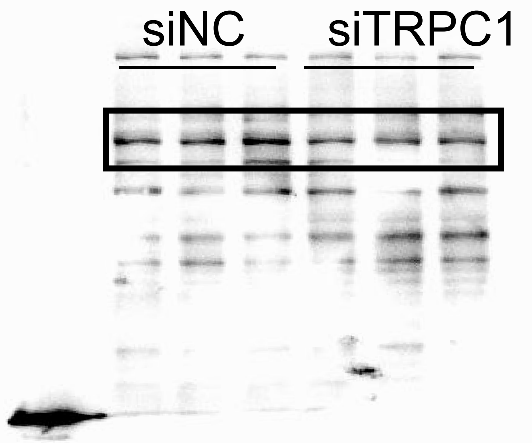

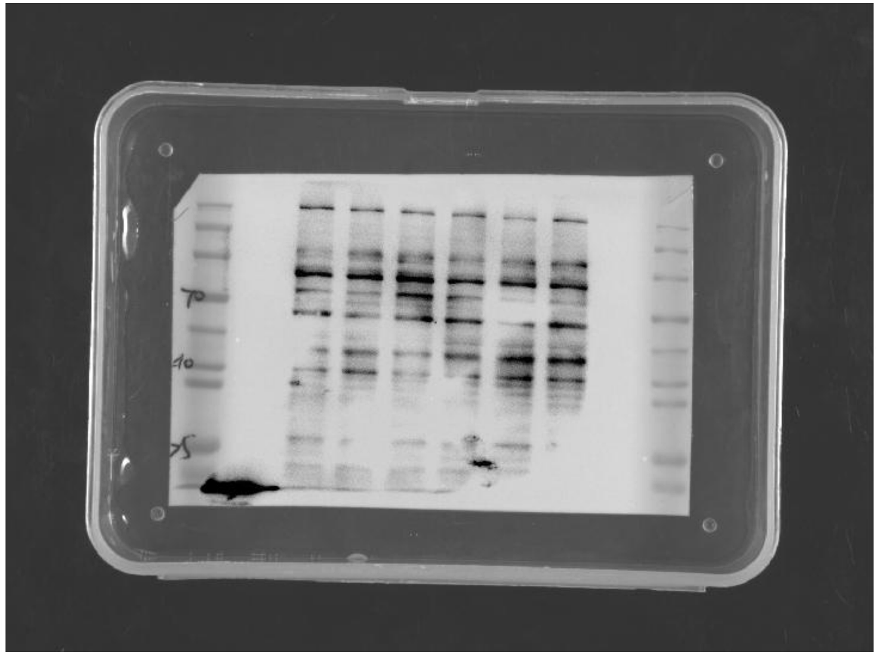

HASMC-actin

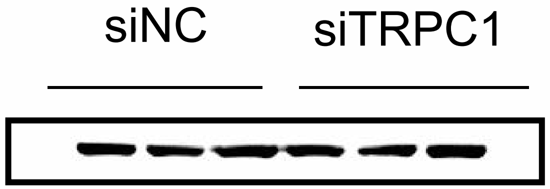

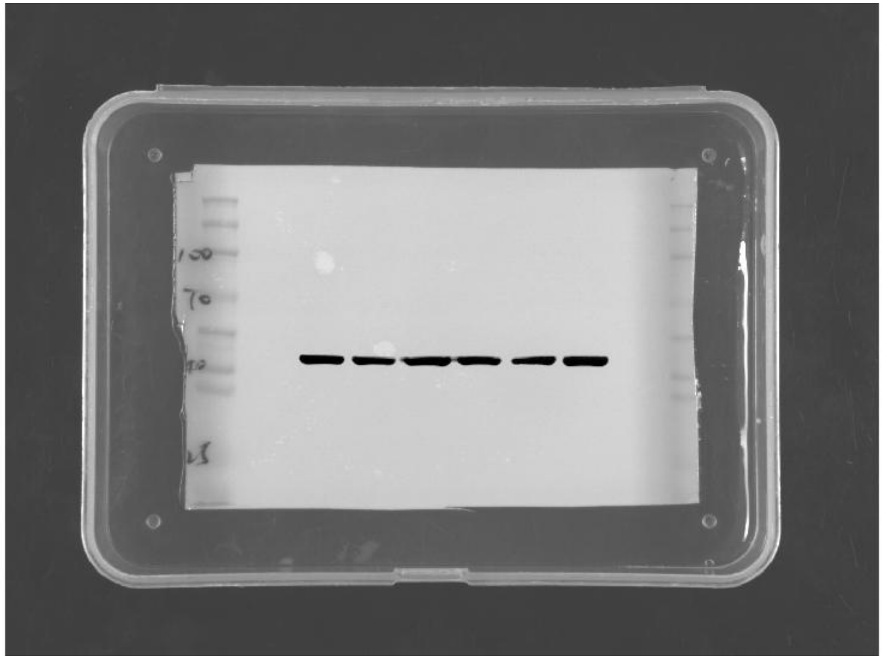

Supplement: Supplementary file 1 [file biomolecules-16-00741-s001.zip › Western Blot Original Images.pdf]
